# CD8^+^ T-cell plasticity regulates vascular regeneration in type-2 diabetes

**DOI:** 10.7150/thno.40663

**Published:** 2020-03-04

**Authors:** Cai Liang, Kevin Y. Yang, Vicken W. Chan, Xisheng Li, Tiffany H.W. Fung, Yalan Wu, Xiao Yu Tian, Yu Huang, Ling Qin, James Y.W. Lau, Kathy O. Lui

**Affiliations:** 1 Department of Chemical Pathology, Prince of Wales Hospital, The Chinese University of Hong Kong, Hong Kong SAR, China.; 2 Faculty of Medicine, The Chinese University of Hong Kong, Hong Kong SAR, China.; 3 School of Biomedical Sciences, The Chinese University of Hong Kong, Hong Kong SAR, China.; 4 Institute of Vascular Medicine, The Chinese University of Hong Kong, Hong Kong SAR, China.; 5 Li Ka Shing Institute of Health Sciences, Prince of Wales Hospital, The Chinese University of Hong Kong, Hong Kong SAR, China.; 6 Department of Orthopaedics and Traumatology, Prince of Wales Hospital, The Chinese University of Hong Kong, Hong Kong SAR, China; 7 Department of Surgery, Prince of Wales Hospital, The Chinese University of Hong Kong, Hong Kong SAR, China

**Keywords:** CD8^+^ T-cells, T-cell plasticity, CD8 checkpoint blockade, vascular regeneration, type-2 diabetes, single cell RNA-seq

## Abstract

In this study, we observe that the ischemic tissues of type-2 diabetic (T2D) patients and mice have significantly more CD8^+^ T-cells than that of their normoglycemic counterparts, respectively. However, the role of CD8^+^ T-cells in the pathogenesis of diabetic vascular complication has been less studied.

**Methods**: We employed loss-of-function studies in mouse models using the non-lytic anti-CD8 antibody that blocks tissue infiltration of CD8^+^ T-cells into the injured tissue. We also performed genome-wide, single-cell RNA-sequencing of CD8^+^ T-cells to uncover their role in the pathogenesis of diabetic vascular diseases.

**Results**: The vascular density is negatively correlated with the number of CD8^+^ T-cells in the ischemic tissues of patients and mice after injury. CD8^+^ T-cells or their supernatant can directly impair human and murine angiogenesis. Compared to normoglycemic mice that can regenerate their blood vessels after injury, T2D mice fail in this regeneration. Treatment with the CD8 checkpoint blocking antibody increases the proliferation and function of endothelial cells in both Lepr^db/db^ mice and diet-induced diabetic *Cdh5-Cre;Rosa-YFP* lineage-tracing mice after ischemic injury*.* Furthermore, single-cell transcriptomic profiling reveals that CD8^+^ T-cells of T2D mice showed a *de novo* cell fate change from the angiogenic, tissue-resident memory cells towards the effector and effector memory cells after injury. Functional revascularization by CD8 checkpoint blockade is mediated through unleashing such a poised lineage commitment of CD8^+^ T-cells from T2D mice.

**Conclusion**: Our results reveal that CD8^+^ T-cell plasticity regulates vascular regeneration; and give clinically relevant insights into the potential development of immunotherapy targeting vascular diseases associated with obesity and diabetes.

## Introduction

Macrovascular disease such as coronary artery disease and peripheral artery disease (PAD) is a leading cause of death in patients with diabetes. The pathogenesis of PAD in diabetic and normoglycemic patients is not the same; and the latter often gains more research attention. Nevertheless, mediators including proangiogenic factors that have been demonstrated to promote post-ischemic revascularization in normoglycemic animal models fail to recapitulate the same effect in diabetic patients 1. Further investigations are needed to understand the pathological difference of diabetic and normoglycemic PAD; and to uncover more novel treatment options targeting diabetic PAD.

Upon injury, new blood vessels are regenerated through sprouting angiogenesis that is tightly linked to local immune responses. Accumulating evidence reveals that infiltrating T-cells of the ischemic tissues facilitate vascular regeneration by providing proangiogenic factors such as vascular endothelial growth factor (VEGF); but athymic nude mice that are devoid of both CD4^+^ and CD8^+^ T-cells demonstrate mitigated regeneration after injury 2. Moreover, post-ischemic revascularization is also impaired in CD4 3- or CD8a 4-deficient mice after injury. More recently, it has been demonstrated that CD4- or T-cell receptor-knockout mice develop tumors that are more abnormally vascularized; yet activation of T-cells particularly the IFN-γ-expressing CD4^+^ T-cells through immune checkpoint blockade enhances blood vessel normalization and perfusion 5.

Nevertheless, obese adipose tissues of both rodents and humans show increased infiltration of both CD4^+^ and CD8^+^ T-cells 6-9 that are known to facilitate the development of insulin resistance 9. The differential roles of these T-cell subsets in regulating diabetic vascular regeneration remain poorly understood. We have previously demonstrated that endothelial cells (ECs) are more inflammatory in type-2 diabetic (T2D) mice; and CD4^+^ Th1 cells impair while CD4^+^FOXP3^+^ regulatory T-cells (Treg) promote vascular regeneration in T2D 10. Following injury, whether CD8^+^ T-cells also regulate the regeneration of peripheral artery system in obesity and diabetes have not been studied.

Here, we examine whether CD8^+^ T-cells of the ischemic tissues play any role in regulating the function and regeneration of blood vessels after injury. We blocked the ability of CD8^+^ T-cells to respond to autoantigens after injury through the CD8 checkpoint blockade previously used to promote antigen-specific immune tolerance in conjunction with the CD4 checkpoint blockade in transplantation settings 11, 12. The CD8 checkpoint blockade led to increased proliferation and function of vascular ECs. Compared to CD8^+^ T-cells that showed an angogenic, tissue resident memory phenotype in normoglycemic mice, we demonstrate that CD8 blockade unleashed the skewed lineage commitment of CD8^+^ T-cells from the effector and effector memory phenotype in T2D mice. The present study provides the first evidence that CD8^+^ T-cells were activated after injury, presumably by recognition of autoantigens, and their plasticity was essential in the regulation of vascular function and regeneration in T2D.

## Experimental Procedures

### Human patients

All procedures were approved by the Clinical Research Ethics Committee of The Chinese University of Hong Kong (CUHK)-New Territories East Cluster Hospitals. Patient samples were collected after below knee amputations from the gastrocnemius muscles of normoglycemic and T2D patients with PAD as previously described 10. For the collection of peripheral blood mononuclear cells (PBMCs), fresh human buffy coat was obtained from healthy volunteers of the Hong Kong Red Cross Blood Transfusion Service. PBMCs were isolated through density gradient centrifugation in Ficoll-Paque® (GE Healthcare Life Sciences) per manufacturer's instructions.

### Mice

All procedures were approved by CUHK Animal Experimentation Ethics Committee. Adult male C57Bl/6, Lepr^db/+^, Lepr^db/db^ and *Cdh5-Cre* were purchased from Jackson Laboratory. High-fat diet (60% fat, Envigo) was fed for 3 months to induce diet-induced obesity (DIO). Glucose tolerance test (GTT) was performed with D-glucose (2 g/kg body weight) injected intraperitoneally (i.p.) following 16 hours of fasting.

### Severe hindlimb ischemia

Mice were anesthetized with Ketamine (100 mg/kg) and Xylasine (10 mg/kg). Unilateral ischemia was induced as previously described 10 by ligating the femoral artery at two points proximal and distal to the bifurcation of superficial and deep femoral artery followed by excision of the intervening segment.

### Administration of mAb

The non-lytic anti-CD8 monoclonal antibody (mAb, clone YTS105) was generated as described previously 11, 12. 0.4 mg IgG2a (isotype control) or anti-CD8 mAbs were i.p. injected once weekly for 4 weeks after induction of ischemia.

### Skeletal muscle/single-fiber isolation

Quantification of EC density and immune infiltrates was examined after single-fiber isolation as described previously 10. For mice, muscles of the femur were minced and enzymatically digested in buffer D containing 800 U/ml collagenase II (Worthington) and 1% Pen/Strep (Gibco) in F10 medium (Sigma) at 37°C for 1.5 hours with agitation. Muscle cells were washed with 10% horse serum (Gibco) and 1% Pen/Strep (Gibco) in F10 medium; and further digested with 11 U/ml dispase (Gibco) and 1000 U/ml collagenase II at 37°C for 0.5 hour with agitation. For patients, gastrocnemius muscles were minced and digested in buffer D. Two rounds of agitated digestion were needed with each at 37°C for 1 hour.

### Primary EC isolation

Mouse ECs were isolated from the lung tissues of 5-week old C57Bl/6 mice as described previously 13. Briefly, murine lung tissues were removed aseptically, rinsed in phosphate-buffered saline (PBS), minced into ~1x2 mm^2^, and digested in 20 ml 400 U/ml collagenase II and 5.5 U/ml dispase at 37°C for 45 minutes with agitation. After that, the suspension was washed twice in EC growth medium (EGM-2, Lonza) and the cell pellet was resuspended and seeded into T25 flask for differential plating. After 1 hour of incubation, the supernatant containing non-ECs was removed and replaced with fresh EGM-2 medium.

### Cell cultures

Naïve CD45^+^CD3^+^CD8^+^ T-cells were purified from the spleen of C57Bl/6 mice by flow cytometry; and activated *in vitro* by anti-CD3 (Biolegend), anti-CD28 (Biolegend) and 50 ng/ml IL-2 (Peprotech) for 3 days. After that, T-cells were co-cultured with mouse ECs in a ratio of 1:1 EC:T-cells as described previously 10. Mouse ECs were cultured for 3 days with T-cells or T-cell conditioned medium in 1:1 EGM-2 medium and T-cell medium containing RPMI 1640, 10 mM HEPES and 1 mM sodium pyruvate supplemented with 25 mM L- or D-glucose (Sigma). Human CD45^+^CD3^+^CD8^+^ T-cells were isolated from PBMCs by flow cytometry; and activated by anti-CD3, anti-CD28 and 50 ng/ml IL-2 for 3 days, followed by 50 ng/ml phorbol-12-myristate-13-acetate (Sigma) and 1 μg/ml ionomycin (Sigma) for an additional day. Human endothelial cells (hESC-ECs) were derived from the H9 human embryonic stem cells (hESCs, WiCell). hESCs were maintained in mTseR1 medium (Stemgent) and differentiated into hESC-ECs as previously reported 10. Mature hESC-ECs were cultured in 25 mM L- or D- glucose with the last 3 days being in the presence of T-cells or conditioned medium in the ratio of 1:1 hESC-ECs:T-cells.

### Tube formation assay

25,000 murine lung ECs or 15,000 hESC-ECs were plated onto each well of a 96 well-plate with 50 μl Matrigel (BD). Images were taken after 7-8 hours of cultures and analyzed by Image J with the Angiogenesis Analyzer plugin for quantification of tube networks and total tubule length.

### Flow cytometry and cell sorting

Red blood cells were first removed with lysis buffer (eBiosciences) at room temperature for 5 min. For human tissues, the dissociated mixtures of muscle cells and immune infiltrates or ECs were blocked with 2% serum and incubated with fluorochrome- conjugated antibodies at 4°C for 30 minutes. For mouse tissues, the dissociated mixture of muscle cells, ECs and immune infiltrates were first incubated with 2% rat serum, followed by staining with fluorochrome-conjugated antibodies at 4°C for 30 minutes. For intracellular staining, dissociated cells were re-stimulated *in vitro* at 37°C for 6 hours in presence of 1 μg/ml ionomycin, 50 ng/ml phorbol- 12-myristate-13-acetate and 1X Golgiplug (BD Biosciences). After that, intracellular staining was done by a staining kit per manufacturer's instructions (eBiosciences). We targeted the following antigens in mouse (m) or human (h) tissues: CD3 (m/h), CD8 (m/h), CD31 (m/h), CD45 (m/h), CD144 (h), IFNγ (m), TNFα (m), granzyme B (m) (all from BD Biosciences or Biolegend) and perforin (m, Thermo Scientific). Cells were then washed three times with 2% FBS-containing PBS and analyzed on the flow cytometer (BD FACSAria^TM^ Fusion). Propidium iodide (PI, BD Pharmingen) positive dead cells were excluded from live cell analysis/sorting; and FACS data was analyzed by the FlowJo software (Tree star). Since the same weight of dissected muscles of Lepr^db/db^ contained more fat than Lepr^db/+^, absolute cell number per gram tissue was also normalized to the same cell number after muscle dissociation during which adipocytes were removed when we compared between Lepr^db/+^ and Lepr^db/db^ 10.

### Immunostaining and confocal microscopy

Briefly, muscles were collected in cold PBS and fixed in 4% paraformaldehyde overnight at 4°C. After washing in PBS, muscles were equilibrated in 30% sucrose at 4°C overnight before freezing in optimal cutting temperature (OCT) compound for cryosectioning. Six micrometer sections were blocked with 0.1% Triton X-100/2% goat serum at room temperature for 1 hour, followed by incubation with primary antibodies (CD31, BD Biosciences; Ki67, Abcam; cCASP3 (Cell signaling technology, 9661); smooth muscle actin (SMA), R&D Systems) at 4°C overnight. Alexa-Fluor-conjugated secondary antibodies (Invitrogen) were used at room temperature for 30 min and nuclear counterstain 4', 6-diamidino- 2-phenylindole (DAPI, Vector Labs) was used at room temperature for 10 min. Slides were mounted with fluorescence mounting medium (Dako) and dried at 4°C overnight in the dark. Fluorescence was detected by confocal microscope (Leica) and analysed by ImageJ (NIH) with quantification by blind cell counting.

### Laser Doppler imaging

Mice were anesthetized and blood perfusion was assessed serially at rest with a laser Doppler perfusion imager (Moon Instruments) at days 7, 14, 21 and 28 following ischemia. Vascularity index as a measure of perfusion (normalized to the muscle area) was quantified and expressed by a ratio of ischemic to non-ischemic limbs as described previously 10.

### Wire myograph

Vasoreactivity was measured in wire myograph as previously described 10, 14. Femoral arteries were removed and dissected in oxygenated ice-cold Krebs solution that contained (mmol/L): 119 NaCl, 4.7 KCl, 2.5 CaCl_2_, 1 MgCl_2_, 25 NaHCO_3_, 1.2 KH_2_PO_4_ and 11 D-glucose. Measurements of isometric tension were recorded in wire myograph (Danish Myo Technology). The arterial segments were stretched to optimal baseline tension at 1 mN. After that, they were washed in Krebs solution for three times and allowed to equilibrate for 1 hour before contracted with 60 mmol/L KCl to test viability. Endothelium- dependent relaxation (EDR) was measured by testing concentration-responses to cumulative addition of acetylcholine (ACh) in phenylephrine (Phe, 3 μmol/L) pre-contracted segments. Some arteries were incubated with nitric oxide (NO) synthase inhibitor N(ω)-Nitro-L-Arginine Methyl Ester (L-NAME, 100 µmol/L, 30 minutes) before testing vasodilation. Vasoconstriction induced by cumulative concentration of Phe was tested before and after L-NAME, which reflects basal NO production.

### Single-cell encapsulation and library preparation

CD45^+^CD3^+^CD8^+^ T-cells used for single-cell RNA-sequencing (scRNA-seq) were dissociated into single cells from the ischemic muscle of Lepr^db/+^ or Lepr^db/db^ mice as aforementioned. Single-cell libraries were prepared with the Chromium Single Cell 3' Reagent Kits v2 (10x Genomics) as previously described 15. Briefly, sorted single cell suspension was prepared as gel beads in emulsion (GEMs) on Single Cell 3' Chips v2 (10x Chromium) using the Chromium Controller (10x Genomics). Barcoded RNA transcripts in each single cell were reverse transcribed within GEM droplets. Complementary DNA (cDNA) was purified with DynaBeads MyOne Silane beads (Invitrogen) and then amplified for subsequent library construction. Sequencing libraries were prepared by fragmentation, end-repair, ligation with indexed adapters, and polymerase chain reaction (PCR) amplification using the Chromium Single Cell 3' library kit v2 (10x Genomics). Nucleic acid was cleaned up after each step using SPRIselect beads (Beckman Coulter). Libraries were then quantified by Qubit and real-time quantitative PCR on a LightCycler 96 System (Roche).

### scRNA-seq and functional annotations

Pooled libraries were sequenced on the Illumina HiSeq 2500 platform with a customized paired-end dual index format (98/26/0/8 bp) according to manufacturer's instructions. Data were processed, aligned, and quantified using the Cell Ranger Single-Cell Software Suite (v 2.1.1). Briefly, data were demultiplexed based on the 8 base-pair sample index, 16 base-pair Chromium barcodes, and 10 base-pair unique molecular identifiers (UMI). Data were then aligned on *Mus musculus* Cell Ranger transcriptome reference (mm10, v.1.2.0) for gene expression quantification. Cells were filtered out from subsequent analysis if their mRNA contents were above or below two standard deviations of the mean value; if they showed high mitochondrial contents as demonstrated by fractions of mitochondrial transcripts > 10%; or if they did not express the *Cd3* transcript. *t*-SNE plots and overexpressed gene identification were performed according to the pipeline of Cell Ranger Single-Cell Sofrware Suite (v.2.1.1). Trajectory analysis was performed by applying Monocle 2 on genes with high dispersion (dispersion_empirical >= 1.5* dispersion_fit). Branched Expression Analysts Modeling (BEAM) in Monocle 2 was performed to identify branch-specific differentially expressed genes with q-value cutoff of 0.01. Differentially expressed genes were clustered by hierarchical clustering analysis and further annotated with GO enrichment analysis using database for annovation, visualization and integrated discovery (DAVID) Bionformatics Resources (v6.8).

### Real-time quantitative PCR (qPCR)

Total RNA was isolated from flow cytometer- sorted live cells using RNeasy mini kit (Qiagen) and reverse transcribed using iScript cDNA synthesis kit (Bio-Rad), according to manufacturers' instructions. qPCR was analysed on a Read-Time PCR system (Bio-Rad) via SYBR Green (Bio-Rad). Gene expression was indicated by a relative quantitation value determined by the 2_ddCT method, which represents the fold change normalized to housekeeping genes, beta-actin and Gapdh. The relative gene expression level of each sample was compared with an internal control.

### Statistics

All data were expressed as mean ± standard error of mean (SEM) with biological replicates, n=6 unless otherwise specified, performed under the same conditions. Statistical analysis was performed using the 2-sided unpaired Student's *t* test for comparing differences between two groups; while data from over two groups was analyzed by one-way ANOVA followed by Tukey's method for multiple comparisons. Quantification of tube networks and total tubule length was analysed by a one-way analysis of variance (ANOVA) followed by Bonferroni's post-hoc tests when comparing each parameter with the PBS control. Significance was accepted when *P* < 0.05. Correlation between two variables was determined by the Pearson correlation coefficient calculated using R.

## Results

### Human CD8^+^ T-cells impair endothelial cell proliferation and survival in hyperglycemia

**Figure 1:**
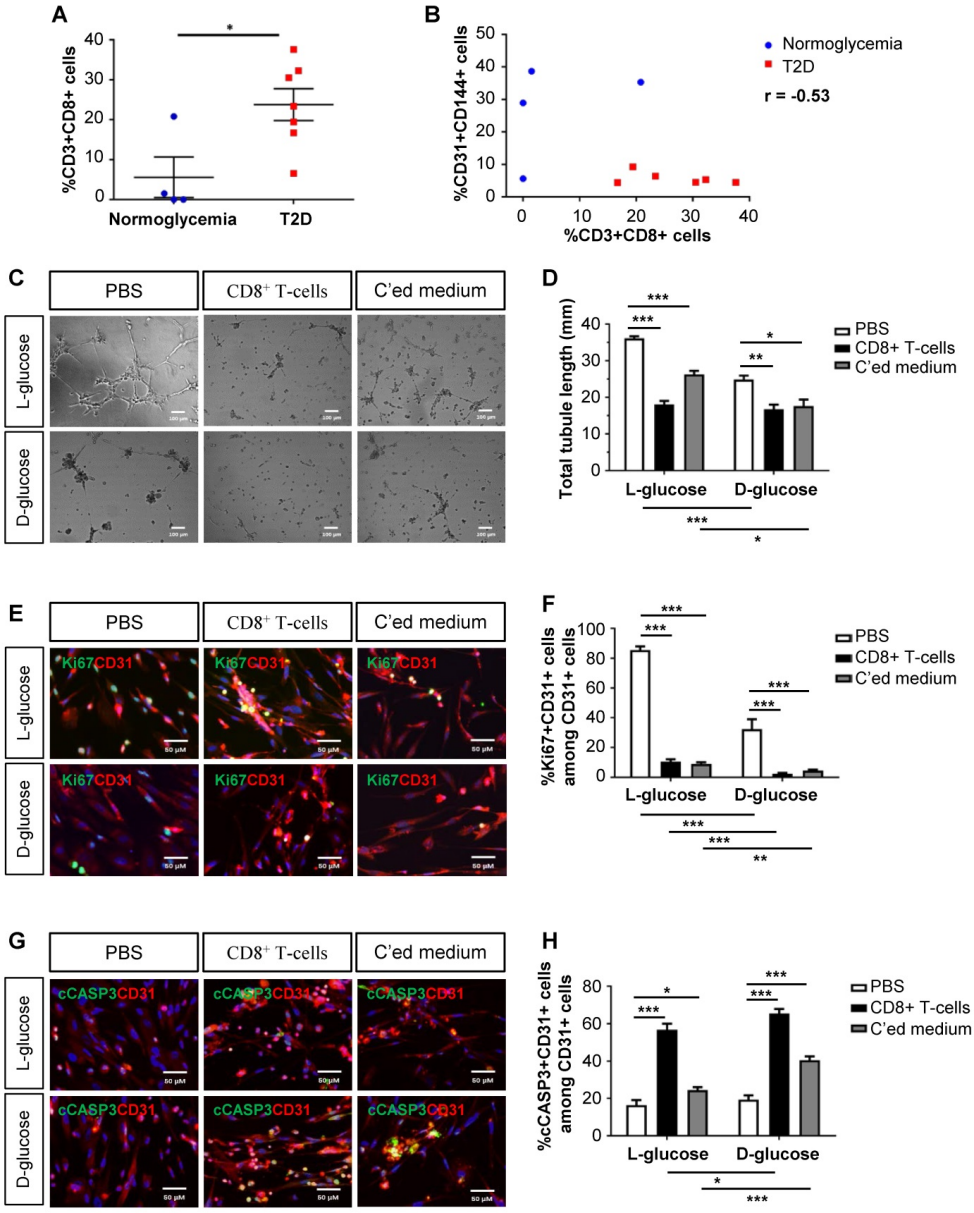
** Human CD8^+^ T-cells impair endothelial cell proliferation and survival in hyperglycemia.** (A) Flow cytometric quantification of %CD3^+^CD8^+^ cells in the ischemic muscles of normoglycemic (n=4) and T2D (n=7) patients with PAD, respectively. (B) Scatter plots showing a negative correlation between %CD31^+^CD144^+^ ECs and %CD3^+^CD8^+^ T-cells in the ischemic muscles of normoglycemic and T2D patients. (C) *In vitro* tube formation assay, scale bars: 100 um; and (D) quantification of total tubule length of hESC-ECs cultured in normoglycemic (25 mM L-glucose) or hyperglycemic (25 mM D-glucose) condition with CD3^+^CD8^+^ sorted cells and their conditioned medium, respectively. (E, G) Immunostaining and (F, H) quantifications for Ki67^+^ or cCASP3^+^ (green) and CD31^+^ (red) cells with nuclear DAPI counterstain (blue) showing significantly reduced numbers of Ki67^+^CD31^+^ cells and increased numbers of cCASP3^+^CD31^+^ cells among total CD31^+^ hESC-ECs per unit area after cultured in 25 mM D- than L-glucose (n=3 independent experiments), scale bars: 50 μm. (A, B, D, F, H) All data are presented as mean +/- S.E.M., *indicates p<0.05, **p<0.01 and ***p<0.001.

As demonstrated previously, we collected the gastrocnemius muscles and nearby arteries of the amputated limbs of normoglycemic and T2D patients with PAD, respectively 10. When we reanalysed the data, we found that there were significantly increased %CD3^+^CD8^+^ T-cells in the ischemic tissues of T2D than normoglycemic patients (Figure 1A). Statistical analysis revealed a negative correlation between the vascular density and tissue infiltration of CD8^+^ T-cells in the ischemic tissues of these patients (Figure 1B, Pearson's correlation coefficient r = -0.53). Although it has been reported that CD8^+^ T-cells are required for neovascularization after ischemic injury in normoglycemic patients 4, we hypothesized that too many CD8^+^ T-cells such as in the case of T2D could be detrimental to EC function and survival.

We cultured mature hESC-ECs in 25 mM L- or D-glucose with solvent control (PBS), human CD8^+^ T-cells and their conditioned medium, respectively (Figure 1C-H). CD8^+^ T-cells or their conditioned medium significantly reduced the total tubule length of ECs compared to that of the control in both L- and D-glucose, respectively (Figure 1C-D). To ask if CD8^+^ T-cells impaired angiogenesis by reducing EC proliferation or survival, we performed co-staining for specific markers of ECs, CD31, and proliferation, Ki67 (Figure 1E), or apoptosis, cleaved caspase 3 (cCASP3, Figure 1G). Our results showed that CD8^+^ T-cells or their conditioned medium significantly reduced %Ki67^+^CD31^+^ (Figure 1F) and increased %cCASP3^+^CD31^+^ (Figure 1H) ECs compared to that of the control. Moreover, it appeared that direct contact with CD8^+^ T-cells was more efficient than the paracrine effect of conditioned medium in promoting EC apoptosis (Figure 1H). Furthermore, D-glucose alone could significantly impede tube formation (Figure 1D) and inhibit proliferation (Figure 1F) of ECs compared to L-glucose. The capability of CD8^+^ T-cells or their conditioned medium in inhibiting proliferation (Figure 1F) or promoting apoptosis (Figure 1H) of ECs was also enhanced in D- than L-glucose, suggesting that CD8^+^ T-cells and D-glucose could have a synergistic effect in regulating angiogenesis.

### Murine CD8^+^ T-cells impair endothelial cell proliferation and survival in hyperglycemia

**Figure 2:**
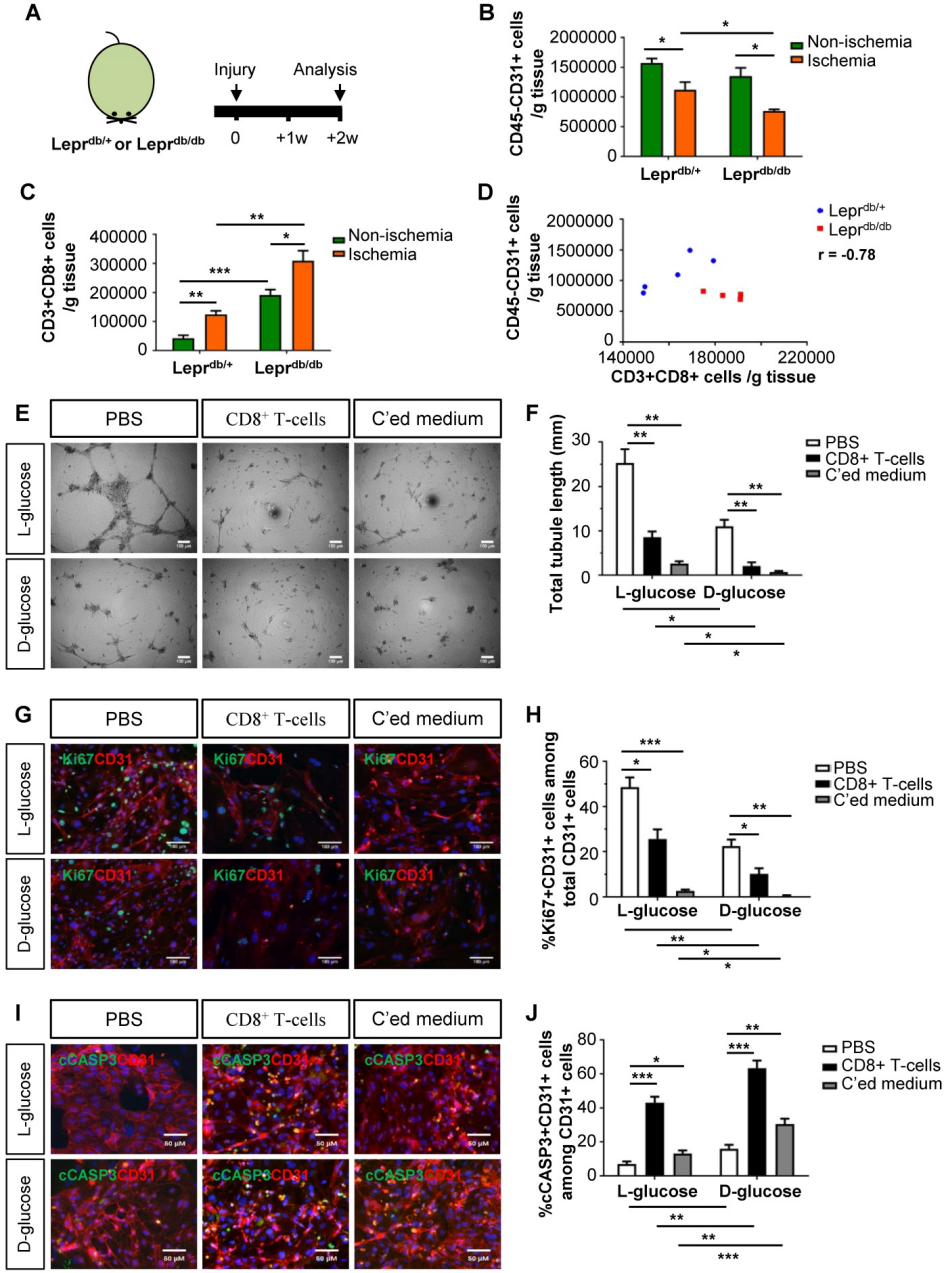
** Murine CD8^+^ T-cells impair endothelial cell proliferation and survival in hyperglycemia.** (A) Schematic diagram showing experimental design. Flow cytometric quantification of the absolute numbers of (B) CD45^-^CD31^+^ and (C) CD3^+^CD8^+^ cells in the ischemic and non-ischemic muscles of Lepr^db/+^ and Lepr^db/db^ mice (n=5 per group) at 2 weeks after injury, respectively. (D) Scatter plots showing a negative correlation between %CD45^-^CD31^+^ ECs and %CD3^+^CD8^+^ T-cells in the ischemic muscles of Lepr^db/+^ and Lepr^db/db^ mice. (E) *In vitro* tube formation assay, scale bars: 100 um; and (F) quantification of total tubule length of muring lung ECs cultured in normoglycemic (25 mM L-glucose) or hyperglycemic (25 mM D-glucose) condition with CD3^+^CD8^+^ sorted cells and their conditioned medium, respectively. (G, I) Immunostaining and (H, J) quantifications for Ki67^+^ (green, scale bar: 100 μm) or cCASP3^+^ (green, scale bar: 50 μm) and CD31^+^ (red) cells with nuclear DAPI counterstain (blue) showing significantly reduced numbers of Ki67^+^CD31^+^ cells and increased numbers of cCASP3^+^CD31^+^ cells among total CD31^+^ lung ECs per unit area after cultured in 25 mM D- than L-glucose (n=3 independent experiments). (B-D, F, H, J) All data are presented as mean +/- S.E.M., *indicates p<0.05, **p<0.01 and ***p<0.001.

We then examined the role of CD8^+^ T-cells in the pathogenesis of PAD in T2D using mouse models. We utilized the control Lepr^db/+^ and T2D Lepr^db/db^ mice; and analysed data at 2 weeks after induction of hindlimb ischemia (Figure 2A). We observed a significantly reduced number of CD45^-^CD31^+^ ECs (Figure 2B) and increased number of CD3^+^CD8^+^ T-cells (Figure 2C) in the ischemic muscles of Lepr^db/db^ than Lepr^db/+^ mice. We also demonstrated a negative correlation between the vascular density and tissue infiltration of CD8^+^ T-cells in the ischemic muscles (Figure 2D, r = -0.78).

To ask if CD8^+^ T-cells can directly impair murine angiogenesis, we cultured murine lung ECs in 25 mM L- or D-glucose with solvent control (PBS), murine CD8^+^ T-cells and their conditioned medium, respectively (Figure 2E-J). CD8^+^ T-cells or their conditioned medium significantly reduced the total tubule length of ECs compared to that of the control in both L- and D-glucose, respectively (Figure 2E-F). We also performed co-staining for CD31, Ki67 and cCASP3 (Figure 2G, I). CD8^+^ T-cells or their conditioned medium significantly reduced %Ki67^+^CD31^+^ (Figure 2H) and increased %cCASP3^+^CD31^+^ (Figure 2J) ECs compared to the control. Moreover, direct contact with CD8^+^ T-cells was more efficient than the conditioned medium in promoting EC apoptosis (Figure 2J). Furthermore, D-glucose alone could significantly impede tube formation (Figure 2F), inhibit proliferation (Figure 2H) and promote apoptosis (Figure 2J) of ECs compared to L-glucose. The capability of CD8^+^ T-cells or their conditioned medium in inhibiting proliferation (Figure 2H) or promoting apoptosis (Figure 2J) of ECs was enhanced in D- than L-glucose. Altogether, our findings were in line with the human data that CD8^+^ T-cells impaired EC proliferation and survival in T2D.

### CD8 checkpoint blockade potentiates endothelial cell proliferation in Lepr^db/db^ mice after injury

**Figure 3:**
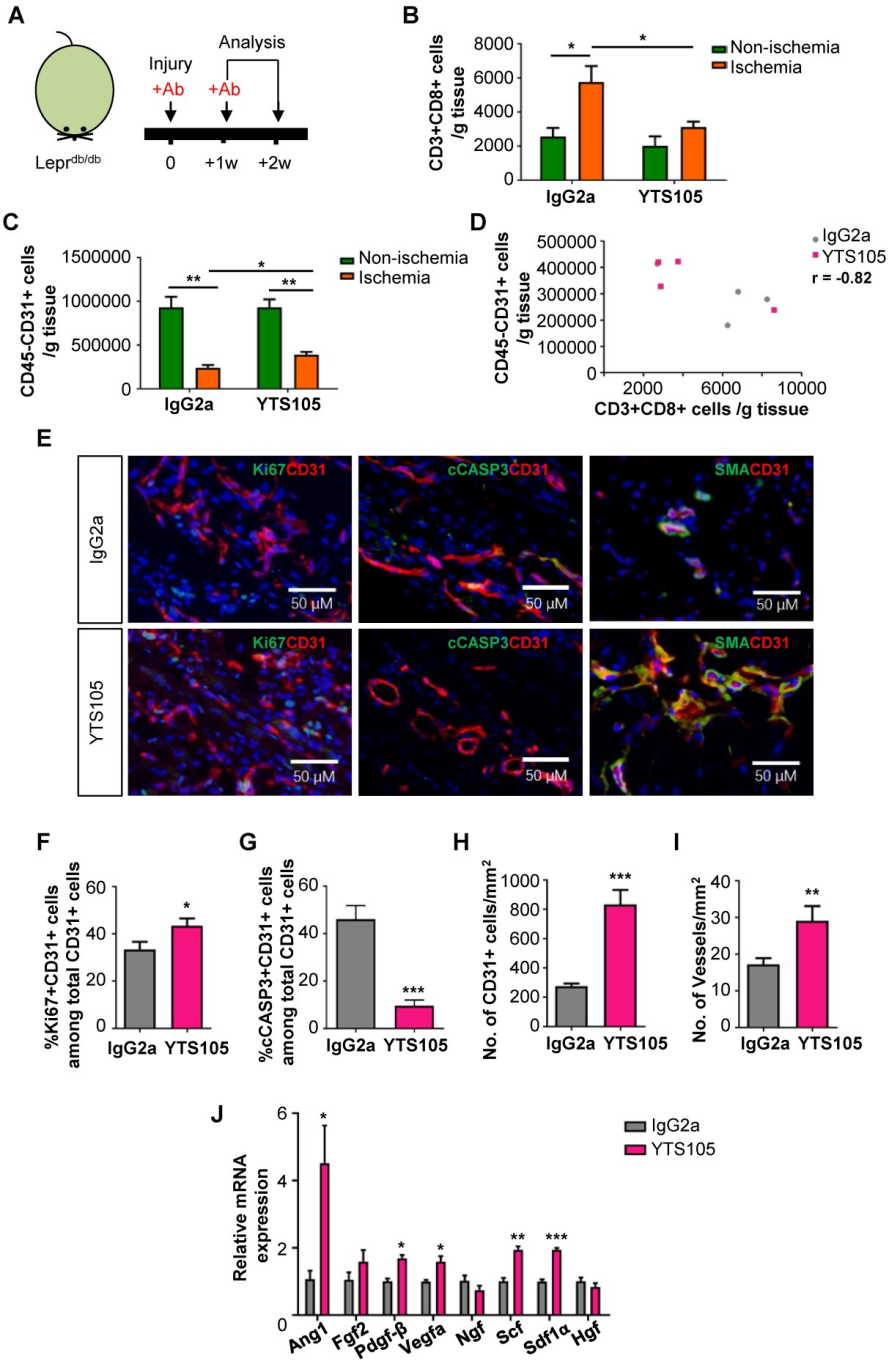
** CD8 checkpoint blockade potentiates endothelial cell proliferation in Lepr^db/db^ mice after injury.** (A) Schematic diagram showing experimental design. Flow cytometric quantification of the absolute numbers of (B) CD3^+^CD8^+^ and (C) CD45^-^CD31^+^ cells in the ischemic and non-ischemic muscles of IgG2a- or YTS105-treated Lepr^db/db^ mice (n=4-5 per group) at 2 weeks after injury, respectively. (D) Scatter plots showing a negative correlation between CD45^-^CD31^+^ ECs and CD3^+^CD8^+^ T-cells in the ischemic muscles of IgG2a- or YTS105-treated mice. (E) Immunostaining on frozen sections and quantification for Ki67^+^ (green), cCASP3^+^ (green) or SMA^+^ (green) and CD31^+^ (red) cells with nuclear DAPI counterstain (blue) showing (F) significantly increased Ki67^+^CD31^+^ proliferating ECs and (G) reduced cCASP3^+^CD31^+^ apoptotic cells at day 7 after injury; and (H) increased number of CD31^+^ cells and (I) increased number of SMA^+^CD31^+^ blood vessels at day 14 after injury. Results are demonstrated as cells counted per unit area in the ischemic muscles of YTS105- than IgG2a-treated mice, n=5 per group, scale bars: 50 μm. (J) Quantitative RT-PCR of CD45^-^CD31^+^ ECs purified from the ischemic muscles at day 7 after injury by flow cytometry; gene expression levels of the YTS105- are compared to that of IgG2a-treated group, n=3 per group. (B-D, F-I) All data are presented as mean +/- S.E.M., *indicates p<0.05, **p<0.01 and ***p<0.001.

To elucidate the potential effect of CD8 checkpoint blockade in therapeutic angiogenesis in T2D, we blocked the activation, proliferation and/or infiltration of CD8^+^ T-cells with a non-lytic anti-CD8 mAb (clone YTS105) as previously described 11, 12 (Figure 3A). There was significantly reduced infiltration of CD3^+^CD8^+^ T-cells (Figure 3B) and increased CD45^-^CD31^+^ EC density (Figure 3C) in the ischemic muscles of YTS105- than IgG2a- (isotype control) treated Lepr^db/db^ mice with a significant negative correlation coefficient (r = -0.82, Figure 3D) at 2 weeks after injury.

To ask if CD8 checkpoint blockade promoted EC proliferation, we performed immunostaining (Figure 3E) and demonstrated significantly increased %Ki67^+^CD31^+^ cells (Figure 3F) but significantly reduced %cCASP3^+^CD31^+^ cells (Figure 3G) among total CD31^+^ ECs in the ischemic muscles of YTS105- than IgG2a-treated group at day 7 after ischemic injury. Moreover, there were significantly increased numbers of CD31^+^ ECs (Figure 3H) and of SMA^+^CD31^+^ blood vessels (Figure 3I) in the ischemic muscles of YTS105- than IgG2a-treated group at 2 weeks after injury. Furthermore, we performed qPCR and compared the expression levels of angiogenic genes in ECs respectively purified from the ischemic muscles of YTS105- and IgG2a-treated mice at day 7 after injury. We showed significantly upregulated expression levels of *Ang1*, *Pdgfb*, *Vegfa*, *Scf* and *Sdf1a* in ECs of YTS105- than IgG2a-treated group (Figure 3I). Altogether, our findings suggested that CD8 checkpoint blockade increased vascular/capillary density, promoted EC proliferation, reduced endothelial cell death and elevated angiogenic gene expression in the ischemic tissues of T2D mice.

### CD8 checkpoint blockade promotes vascular regeneration and function in Lepr^db/db^ mice after injury

**Figure 4:**
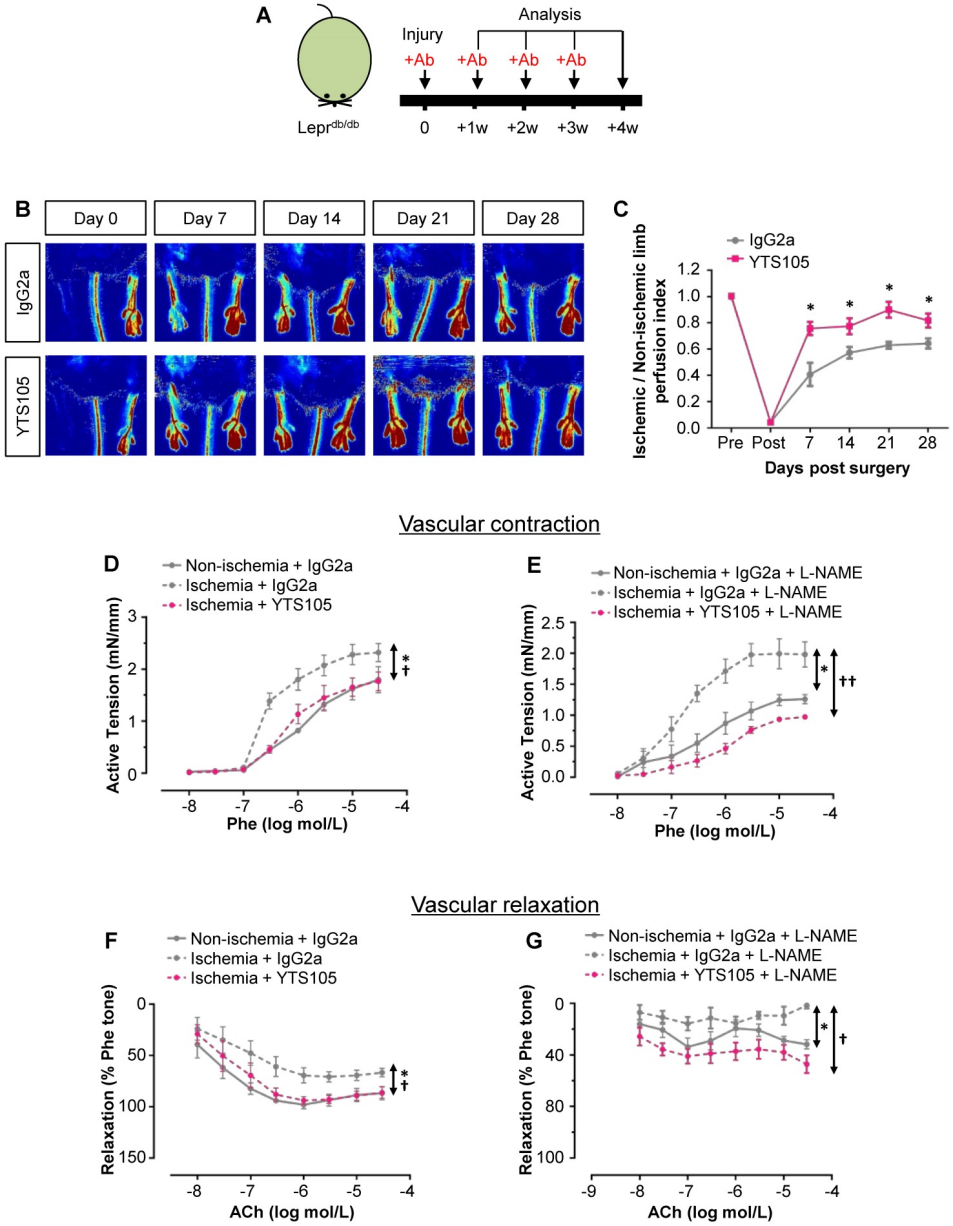
** CD8 checkpoint blockade promotes vascular regeneration and function in Lepr^db/db^ mice after injury.** (A) Schematic diagram showing experimental design. (B) Laser Doppler images and (C) quantification of the ischemic/non-ischemic limb perfusion index showing a time-dependent dynamic change in the blood flow of YTS105- compared to that of IgG2a-treated Lepr^db/db^ mice. (D, E) Concentration-dependent vasocontractions of mouse femoral arteries of the limbs in response to Phe were measured after treatment with IgG2a or YTS105 for 2 weeks, respectively. (F, G) Concentration-dependent EDRs of mouse femoral arteries of the ischemic in response to ACh were measured after treatment with IgG2a or YTS105 for 2 weeks, respectively. (E, G) Reactions were antagonized by co-treatment with NOS inhibitor, L-NAME. (C, D-G) All data are presented as mean +/- S.E.M., n=4-6 per group, *indicates p<0.05; (D-G) *p<0.05 in the ischemic limbs compared to the non-ischemic limbs of IgG2a-treated group; †p<0.05 and ††p<0.01 in the ischemic limbs of YTS105- compared to that of IgG2a-treated group.

To elucidate the role of CD8^+^ T-cells in functional reperfusion, we performed laser Doppler imaging in Lepr^db/db^ weekly for 4 weeks after ischemic injury (Figure 4A). Our results showed a significant improvement of blood reperfusion in the ischemic limbs of YTS105- than IgG2a-treated group (Figure 4B-C). To examine the role of CD8^+^ T-cells in endothelium-dependent vasoreactivity, we performed wire myography in Lepr^db/db^ at 2 weeks after injury. There were significantly reduced phenylephrine (Phe)-mediated vasocontractions in the femoral arteries of ischemic limbs of YTS105- than IgG2a-treated group; and there was no difference in vasocontractions of the ischemic limbs of YTS105-treated group compared to that of the non-ischemic limbs (Figure 4D). Moreover, the same trend could be seen even in the presence of nitric oxide (NO) synthase inhibitor L-NAME (Figure 4E). Similarly, there were also significantly augmented acetylcholine (ACh)-mediated EDRs in the femoral arteries of ischemic limbs of YTS105- than IgG2a-treated group (Figure 4F); and there was no difference in the EDRs of the ischemic limbs of YTS105-treated group compared to that of the non-ischemic limbs, indicating that CD8 checkpoint blockade restored EC function. Although the ACh-mediated ERDs were largely abolished by L-NAME in all the groups, the basal levels of EDRs of the YTS105- were significantly greater than that of the IgG2a-treated group, suggesting better endothelial function following treatment of YTS105. Taken together, our findings showed that CD8 checkpoint blockade rescued vascular function after ischemic injury in T2D mice.

### CD8 checkpoint blockade promotes vascular regeneration and function in DIO mice after injury

In addition to Lepr^db/db^, we also used DIO mice as an independent T2D model to examine the therapeutic potential of CD8 checkpoint blockade in promoting vascular regeneration in T2D mice (Figure S1). Impaired glucose tolerance was confirmed by GTT (Figure S1A-B). Similar to Lepr^db/db^, there was significantly reduced infiltration of CD3^+^CD8^+^ T-cells (Figure S1C) and increased CD45^-^CD31^+^ EC density (Figure S1D) in the ischemic muscles of YTS105- than IgG2a- (isotype control) treated mice with a significant negative correlation coefficient (r = -0.89, Figure S1E) at 4 weeks after injury. To determine if CD8 checkpoint blockade also promoted functional reperfusion in DIO mice, we performed laser Doppler imaging weekly for 4 weeks after ischemic injury (Figure S1F). Consistent with the results of Lepr^db/db^ (Figure 4), we showed a significant improvement in blood reperfusion of the ischemic limbs of YTS105- than IgG2a-treated group (Figure S1G). Since DIO mice were at least 3 months older than Lepr^db/db^ following treatment on high-fat diet before induction of ischemia, we observed a more severe PAD phenotype such as auto-amputation in DIO mice. Nevertheless, auto-amputation was reduced from 80% in the IgG2a- to 20% in the YTS105-treated group (Figure S1H). Collectively, our findings showed that CD8 checkpoint blockade promoted post-ischemic vascular regeneration in two independent T2D models.

To examine if the therapeutic effect of CD8 checkpoint blockade was specific to ECs, we performed fate mapping of ECs via *Cdh5-Cre* in addition to immunostaining for CD31. Following treatment on high-fat diet for 3 months, we analysed data at 2 weeks after ischemic injury in the genetic lineage-tracing *Cdh5-Cre;Rosa26^YFP/+^* mice. Our quantitative results by flow cytometry showed a significant loss of YFP^+^CD31^+^ ECs in the ischemic than non-ischemic muscles of IgG2a- but not YTS105-treated group; and a significant increase in %YFP^+^CD31^+^ ECs in the ischemic muscles of YTS105- than IgG2a-treated group (Figure S2A-B). Notably, there was a significant increase in %YFP^+^CD31^-^ cells in the ischemic than non-ischemic muscles of IgG2a-treated group; and a significant increase in %YFP^+^CD31^-^ cells in the ischemic muscles of IgG2a- than YTS105-treated group (Figure S2A, C). In fact, we reported previously that the YFP^+^CD31^-^ cells of the ischemic muscles of IgG2a-treated group could be immature ECs with reduced/impaired endothelial function 10.

### Genome-wide, single-cell transcriptomic profiling reveals that CD8^+^ T-cells harbor a poised transcriptome in Lepr^db/db^ mice after injury

**Figure 5:**
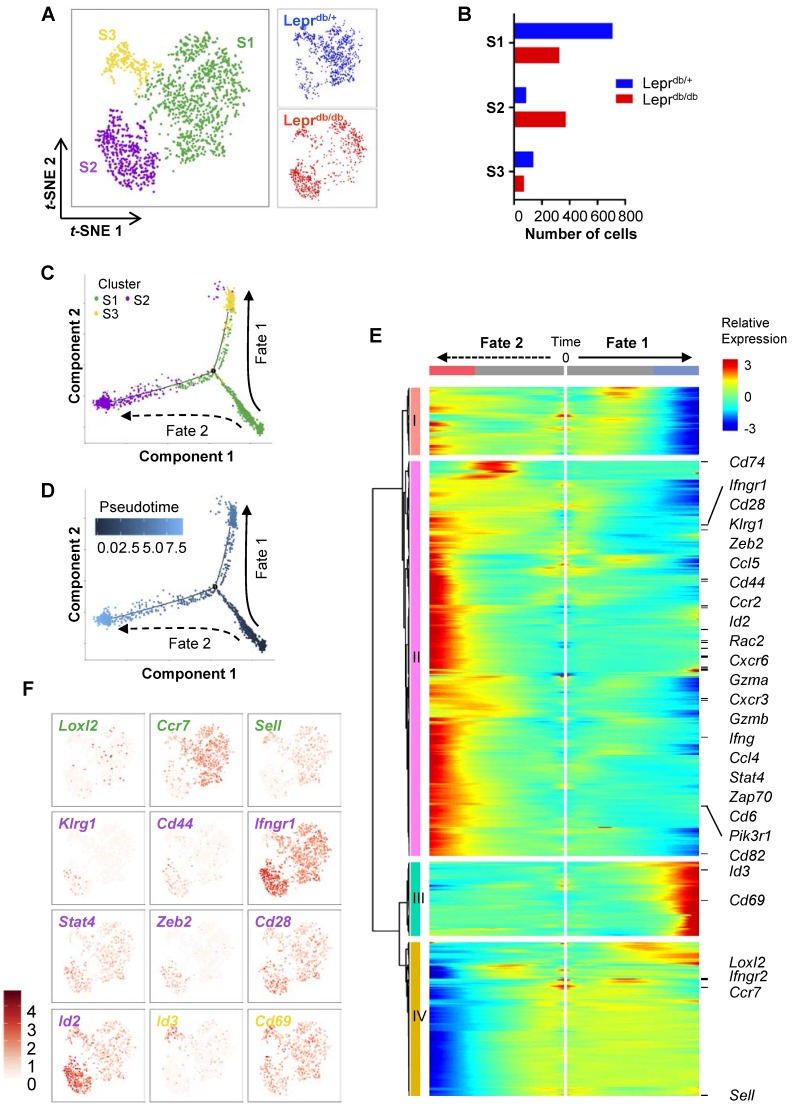
** Genome-wide, single-cell transcriptomic profiling reveals a skewed cell fate commitment of CD8^+^ T-cells in Lepr^db/db^ mice after injury.** (A) Biaxial scatter plots by *t*-SNE analysis showing single-cell transcriptomic clustering of ~ 1,000 CD45^+^CD3^+^CD8^+^ cells purified from the ischemic muscles of Lepr^db/+^ and Lepr^db/db^ mice, respectively, at day 7 after injury by flow cytometry; and three distinct subsets are identified. (B) Distribution of cell number of CD8^+^ T-cells in each cell subset as determined by *t*-SNE. (C, D) Monocle ordering of individual cells showing two branched developmental and response trajectories of CD8^+^ T-cells of Lepr^db/+^ and Lepr^db/db^ mice after ischemic injury in a pseudotime-dependent manner. (E) Heatmap showing branch-dependent genes by BEAM analysis during fate 1 or 2 commitment of CD8^+^ T-cells. A total of 699 genes with significant q-value cutoffs are presented and distinct clusters are identified along the tree. (F) Relative expression levels of specific canonical markers by cells of all three distinct subsets on *t*-SNE plots.

CD8^+^ T-cells are heterogeneous, consisting of naïve, effector, central memory, effector memory and regulatory subsets that vary in location, function and longevity. Previous research has mostly focused on the heterogeneity of CD8^+^ T-cells during viral infections. However, the subsets of CD8^+^ T-cells regulating tissue repair and regeneration have not been studied. To delineate the heterogeneity of CD8^+^ T-cells in non-lymphoid tissues of Lepr^db/+^ and Lepr^db/db^ mice, we performed large-scale, droplet-based single-cell transcriptomic profiling as previously described 15, 16. We purified about ~ 1,000 CD45^+^CD3^+^CD8^+^ T-cells from the ischemic muscles of Lepr^db/+^ and Lepr^db/db^, respectively, at day 7 after injury. After filtering, we obtained 934 and 768 cells from Lepr^db/+^ and Lepr^db/db^, respectively; and performed unsupervised analysis that did not rely on known subset markers. Our *t*-distributed stochastic neighbor embedding (*t*-SNE) analysis revealed three distinct subsets (Figure 5A) that were *Cd3*^+^*Cd4*^-^*Cd8*^+^ (Figure S3). Moreover, the results showed that S1 and S3 were more prominent in Lepr^db/+^ while S2 was the main subset in Lepr^db/db^ (Figure 5B, Table 1).

We then extracted the temporal information from the single-cell profiles and performed unsupervised pseudo-temporal ordering of individual cells using Monocle 17. Monocle ordered cells by progress through differentiation and mapped cells along the pseudotime to reconstruct the developmental and response trajectories of CD8^+^ T-cells of Lepr^db/+^ and Lepr^db/db^ mice after ischemic injury. We found two branched biological processes (Figure 5C); and S1 cells should be of an earlier cell fate that preceded S2 and then S3 cells (Figure 5D). It appeared that S1 cells could be precursor cells that made cell fate decision and subsequently generated alternative lineages, namely S3 cells along fate 1 or S2 cells along fate 2 differentiation. In addition, CD8^+^ T-cells of Lepr^db/+^ mice preferred fate 1 over fate 2; while that of Lepr^db/db^ mice skewed towards fate 2 (Figure S4A).

To implicate genes that could be potential drivers of alternative cell fate commitment of CD8^+^ T-cells of Lepr^db/+^ and Lepr^db/db^ mice after ischemic injury, respectively, branched expression analysis modeling (BEAM) was used to identify genes with branch-dependent expression 18. Heatmap showed 699 genes using q-value cutoffs of 0.01 (Figure 5E, Table S1). Four distinct clusters had been identified along the tree: I and IV showed patterns of low expression at the end points of Fates 1 and 2, respectively; while II and III showed patterns of high expression at the end points of Fates 2 and 1, respectively. Gene ontology analysis of the differentially expressed genes revealed that the most significantly downregulated pathways in cluster I were associated with cell cycle transition and cell proliferation; the most significantly upregulated pathways in cluster II were associated with immune and inflammatory responses, neutrophil chemotaxis and tumor necrosis factor (TNF) production; the most significantly upregulated pathways in cluster III were associated with protein folding and heat response; and the most significantly downregulated pathways in cluster IV were associated with translation (Figure S4B, Table S2). Our results suggested that cells of Fate 1 showed reduced proliferation and increased protein remodelling; while cells of Fate 2 demonstrated increased inflammatory responses with minimal protein translation.

Furthermore, we found that genes of cluster II were overexpressed in S2 cells; genes of cluster III were overexpressed in S3 cells; and genes of cluster IV were overexpressed in S1 cells (Figure 5E). Based on expression of canonical markers, we could further annotate specific CD8^+^ T-cell subsets into angiogenic (*Loxl2*^+^ 19), central memory (*Ccr7*^+^, *Sell*^+^) S1 cells; effector (*Klrg1*^+^, *Cd44*^+^, *Ifngr1*^hi^, *Stat4*^hi^, *Zeb2*^+^ 20,* Id2*^hi^ 21) and effector memory (*Cd28*^hi^ 22, *Id2*^hi^ 23) S2 cells; as well as tissue resident memory (*Id3*^+^ 23, *Cd69*^hi^ 24) S3 cells (Figure 5F). Altogether, we demonstrated that CD8^+^ T-cells differentiated from an angiogeneic, central memory precursor subset to a tissue resident memory subset in Lepr^db/+^ mice (Fate 1), that skewed towards effector and effector memory subsets in Lepr^db/db^ mice (Fate 2) after ischemic injury.

### CD8 checkpoint blockade unleashes the poised commitment of effector CD8^+^ T-cells from Lepr^db/db^ mice after injury

**Figure 6:**
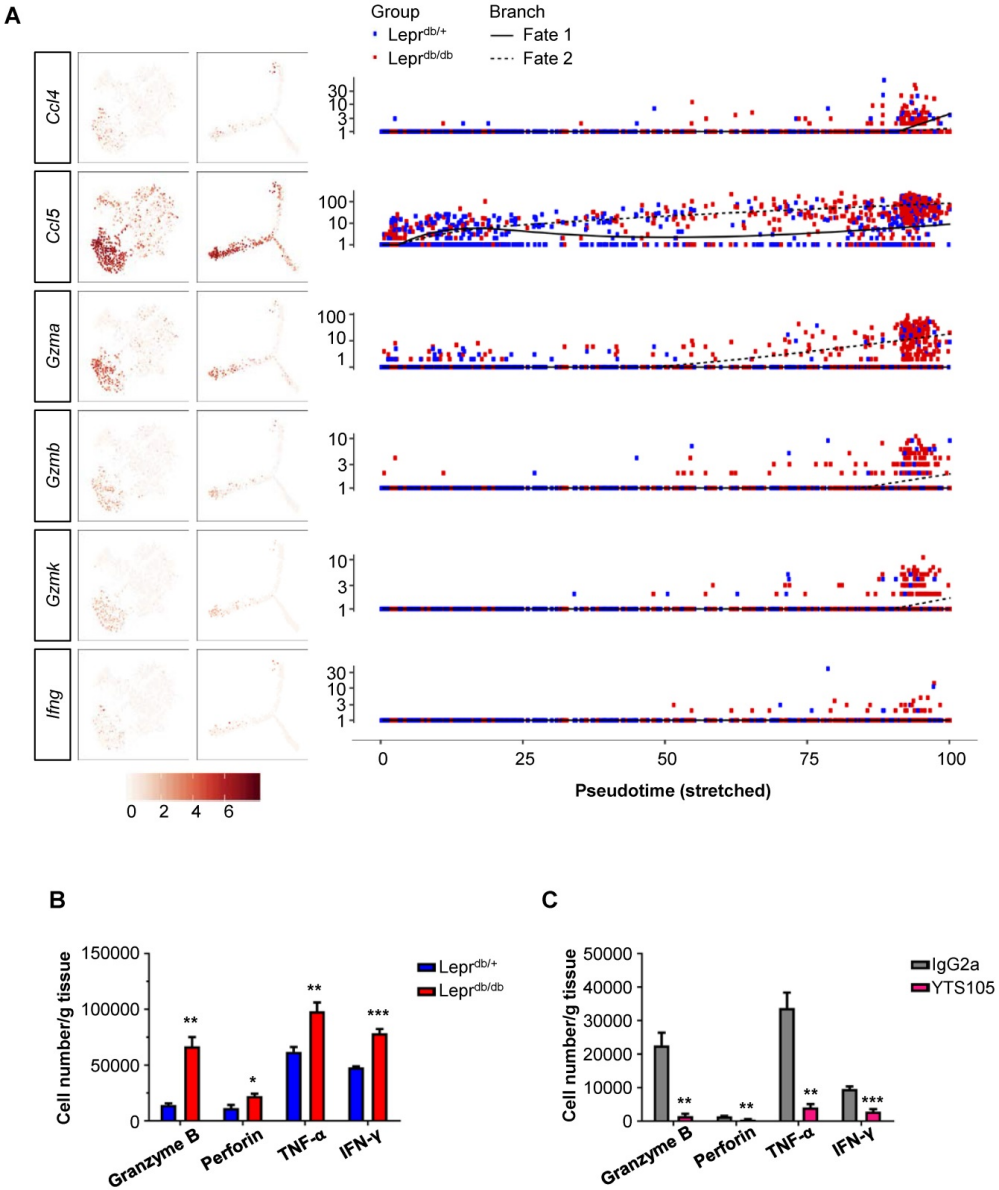
** CD8 checkpoint blockade inhibits the skewed commitment of effector CD8^+^ T-cells in Lepr^db/db^ mice after injury.** (A) Single-cell transcriptomic clustering of ~ 1,000 CD45^+^CD3^+^CD8^+^ cells purified from the ischemic muscles of Lepr^db/+^ and Lepr^db/db^ mice at day 7 after injury, respectively. Biaxial scatter plots showing the relative expression levels of auto/paracrine genes by cells of all three distinct subsets on *t*-SNE and pseudotime plots; and changes in relative expression of individual genes with pseudotime. (B, C) Flow cytometric quantification showing the absolute number per gram tissue of CD8^+^ T-cells with expression of specific cytotoxic granules and cytokines in the ischemic muscles of (B) Lepr^db/+^ and Lepr^db/db^; or (C) IgG2a- and YTS105-treated Lepr^db/db^ mice, respectively, at day 14 after injury. All data are presented as mean +/- S.E.M, n=5 per group, *indicates p<0.05, **p<0.01 and ***p<0.001.

Since the conditioned media of CD8^+^ T-cells directly impaired both human (Figure 1) and murine (Figure 2) angiogenesis, we attempted to determine the paracrine factors upregulated in CD8^+^ T-cells of Lepr^db/db^ mice when compared to that of Lepr^db/+^ mice. Our results showed that *Ccl4*, *Ccl5*, *Gzma*, *Gzmb*, *Gzmk* and *Ifn-g* were preferentially expressed by S2 cells of Lepr^db/db^ mice during fate 2 commitment when compared to that of Lepr^db/+^ mice at day 7 after injury (Figure 6A). We also performed intracellular staining for cytotoxic granules such as granzyme B and perforin; as well as cytokines such as IFN-γ and TNF-α in CD8^+^ T-cells of Lepr^db/+^ and Lepr^db/db^ mice at day 14 after ischemic injury (Figure 6B, S5). Our flow cytometric analysis showed that there were significantly more granzyme B-, perforin-, TNF-α- and IFN-γ-expressing CD8^+^ T-cells of Lepr^db/db^ compared to that of Lepr^db/+^ mice. Moreover, treatment of YTS105 significantly reduced the number of granzyme B-, perforin-, TNF-α- and IFN-γ- expressing CD8^+^ T-cells in the ischemic tissues of Lepr^db/db^ mice compared to that of the IgG2a-treated group (Figure 6C, S6). Taken together, our findings indicated that the CD8 checkpoint blockade promoted vascular regeneration by blocking the skewed commitment of effector CD8^+^ T-cells and inhibiting their production of cytotoxic factors after ischemic injury in T2D mice.

## Discussion

Immune cell-mediated tissue repair has been an emerging paradigm in the field of Regenerative Medicine (for review, see 25-27). Our previous work has also highlighted the importance of peripheral immune cells particularly Treg in facilitating regeneration of the cardiovascular system 10, 16. We demonstrated that CD4^+^ Th1 cells impair while CD4^+^FOXP3^+^ Treg promote vascular regeneration after ischemic injury in T2D mice 10. In addition to CD4^+^ T-cells, we observed a CD3^+^CD4^-^ population in the ischemic tissues of PAD patients as well as their analogous mouse models 10. Here, we further demonstrated that there was a significantly increased proportion of CD3^+^CD8^+^ T-cells in the ischemic tissues of T2D mice when compared to that of the normoglycemic controls; and a negative correlation in the amount of CD8^+^ T-cells and vascular density of the ischemic tissues of both human and murine models was observed. A previous study has revealed that the autoreactive CD8^+^ T-cells are required for promoting post-ischemic revascularization in normoglycemic mice 4. Nevertheless, whether the same is true for T2D mice remains unknown.

To address this question, we blocked infiltration, activation and/or proliferation of CD8^+^ T-cells in the ischemic tissues of T2D mice through CD8 checkpoint blockade. If they were essential for vascular regeneration in T2D mice, we would observe impaired angiogenesis and EC function after CD8 checkpoint blockade, similar to the normoglycemic mice. We used two T2D murine models in this study: the transgenic Lepr^db/db^ and DIO mice. There were significantly increased Ki67^+^CD31^+^ proliferating ECs, reduced cCASP3^+^CD31^+^ apoptotic ECs, and elevated SMA^+^CD31^+^ blood vessels in the ischemic tissues of Lepr^db/db^ mice; and increased CDH5^+^CD31^+^ mature ECs in the diet-induced obese *Cdh5-CreER* lineage-tracing mice of the YTS105 group when compared to that of the IgG2a control group. Furthermore, laser Doppler imaging revealed an increased blood reperfusion and wire myography demonstrated an enhanced vasoreactivity in the ischemic tissues of Lepr^db/db^ mice after treatment with YTS105. Collectively, our findings suggested that the CD8 checkpoint blockade significantly promoted, rather than reduced, EC proliferation and function after ischemic injury in both Lepr^db/db^ and DIO mice. Therefore, it appeared that tissue-infiltrating CD8^+^ T-cells negatively regulated vascular regeneration and EC function in obesity and diabetes.

Since CD8^+^ T-cells displayed an opposite role in vascular regeneration of normoglycemic and T2D mice, we asked if different subsets of CD8^+^ T-cells operate in these mice after injury. To delineate cellular heterogeneity, we performed single cell transcriptomic profiling and found *Ccr7*^+^*Sell* (CD62L)^+^ precursor cells in the ischemic tissues that appeared to be angiogenic as they expressed *Loxl2*, previously reported as a hypoxia-mediated enzyme involved in extracellular matrix remodelling of ECs during sprouting angiogenesis 19. These precursor CD8^+^ T-cells made alternative cell fate decision in Lepr^db/+^ and Lepr^db/db^ mice after injury. As a result, there were less tissue-resident memory cells along fate 1 but more effector/effector memory cells along fate 2 differentiation in Lepr^db/db^ when compared to that of Lepr^db/+^ mice, and *vice versa*.

CD69 is a canonical marker of tissue resident memory CD8^+^ T-cells 24 that are established during resolution of primary infection and provides an important first line of defence for the peripheral tissues upon exposure to the same or similar antigens (for review, see 28). They have been described in a variety of non-lymphoid organs including mucosal tissue and the skin, liver, kidney, pancreas, heart and brain 28. Our findings showed that these cells were localized in the ischemic muscles after fate 1 differentiation, suggesting that they might also play a role in vascular regeneration in response to autoantigens. Indeed, it has also been found that the tissue resident memory T-cells of the skin have a dual role as they direct both the antimicrobial and pro-tissue repair programs 29. They can simultaneously provide an IL-17-mediated immune response under homeostasis and rapidly turn on tissue repair response after injury 29.

We also annotated the effector/effector memory CD8^+^ T-cell identity in the ischemic muscles after fate 2 differentiation based on their characteristics after viral infection. For instance, KLRG1 is a canonical marker of effector but not memory CD8^+^ T-cells 30. It has been reported that *Zeb2* is required for programming CD8^+^ T cells for terminal differentiation in response to viral infection 20. Expression of *Rac2* could facilitate their proliferation as Rac2-deficient T-cells demonstrate reduced proliferation upon stimulation with anti-CD3 or T-cell receptor-specific antigen 31. Moreover, more naïve and memory CD8^+^ T-cells could have been recruited to the ischemic tissues through autocrine secretion of the chemokines *Ccl4* 32 and *Ccl5* 33, respectively. In addition to expression of *Ifng*, *Ifngr1* was upregulated but *Ifngr2* was downregulated in fate 2 cells. Indeed, *Ifngr2* has been reported as downregulated in effector and effector memory CD8^+^ T-cells 34. Expression of *Stat4* in fate 2 cells could facilitate IFN-γ expression following activation by type I interferons as demonstrated in CD8^+^ T-cells during viral clearance 35. Furthermore, expression of the transcription factors *Id3* and *Id2* by fate 1 and 2 cells, respectively, could play a role in their regulation. It has been demonstrated that Id2 is important for the survival of effector cells and formation of short-lived effector memory cells 21, 23; while Id3 is essential for the survival of long-lived memory cells 23.

In addition to CD4^+^ Th1 cells that we previously described 10, the present study added that the skewed commitment of CD8^+^ T-cells towards the effector/effector memory lineage could be detrimental to vascular regeneration after injury in T2D. With findings of these studies, we interpreted the functional relationship of CD4^+^ and CD8^+^ T-cells in modulating vascular regeneration in T2D. There were functional autoreactive CD8^+^ T-cells when the activity of CD4^+^ T-cells was blocked but vascular regeneration was resulted, suggesting that CD8^+^ T-cells needed help from CD4^+^ T-cells for negatively regulating vascular regeneration in T2D. Similarly, there were also CD4^+^ T-cells when the activity of CD8^+^ T-cells was blocked but vascular regeneration was resulted, suggesting that they alone were insufficient for negatively regulating vascular regeneration in T2D.

Taken together, we report the unappreciated role of CD8^+^ T-cells in the pathogenesis of PAD in T2D. Their plasticity might govern the principle outcomes of vascular repair after ischemia with tissue resident memory cells being predominant during vascular regeneration of normoglycemic mice while effector/effector memory cells impaired vascular regeneration of T2D mice. We have also highlighted a hitherto novel role of the CD8 checkpoint blockade in promoting *de novo* functional revascularization by stimulating EC proliferation and function; and by unleashing CD8^+^ T-cells from the effector/effector memory specification in T2D mice. Nevertheless, we did not observe any side effect such as increased risk of infections or changes in the body weight or physical activities after the use of the anti-CD8 mAb systemically. We also demonstrated that the effect of this treatment should be antigen-specific as we only found the localized change of CD3^+^CD8^+^ cells or CD45^-^CD31^+^ cells in the ischemic but not in the non-ischemic limbs of the control IgG2a and YTS105 treated groups after injury, respectively. Therefore, our findings could offer new avenues for developing potential immunotherapy targeting PAD in obesity and diabetes.

## Supplementary Material

Supplementary figures and tables.

## Figures and Tables

**Table 1:** Distribution of cell number of CD8^+^ T-cells in each cell subset as determined by *t*-SNE.

	Total	Lepr^db/+^	Lepr^db/db^
**Total**	1702	934	768
**S1**	1036	710	326
**S2**	457	87	370
**S3**	209	137	72

Cells were purified from the ischemic muscles of Lepr^db/+^ and Lepr^db/db^ mice at day 7 after injury.
