# Supplementary material for: CD8+ T-cell plasticity regulates vascular regeneration in type-2 diabetes
Source: Theranostics. 2020 Mar 4;10(9):4217–32. doi: 10.7150/thno.40663 (PMC7086373; doi:10.7150/thno.40663)
Supplement: Supplementary file 1 — Supplementary figures and tables. [file thnov10p4217s1.pdf]

Supplementary Figures

Figure S1 CD8 checkpoint blockade promotes vascular regeneration and function in DIO mice after injury. (A) Glucose tolerance test showing changes in blood glucose levels with time after intraperitoneal injection of D-glucose in C57 mice fed with normal chow or high fat diet. The high fat diet-fed mice are also known as DIO mice. (B) Quantification of (A) by area under curve (AUC). Flow cytometric quantification of the absolute numbers of (B) $CD3^+CD8^+$ and (C) $CD45^-CD31^+$ cells in the ischemic and non-ischemic muscles of IgG2a- or YTS105-treated DIO mice at 4 weeks after injury, respectively. (D) Scatter plots showing a negative correlation between $CD45^-CD31^+$ ECs and $CD3^+CD8^+$ T-cells in the ischemic muscles of IgG2a- or YTS105-treated DIO mice. (F) Laser Doppler images and (G) quantification of the ischemic/non-ischemic limb perfusion index showing a time-dependent dynamic change in the blood flow of YTS105- compared to that of IgG2a-treated DIO mice. (H) Quantification of autoamputated limbs post-ischemic injury in IgG2a- or YTS105-treated mice. In this figure, all data are presented as mean +/- S.E.M, n=5 per group, *indicates $p<0.05$, **$p<0.01$ and ***$p<0.001$.

Figure S1

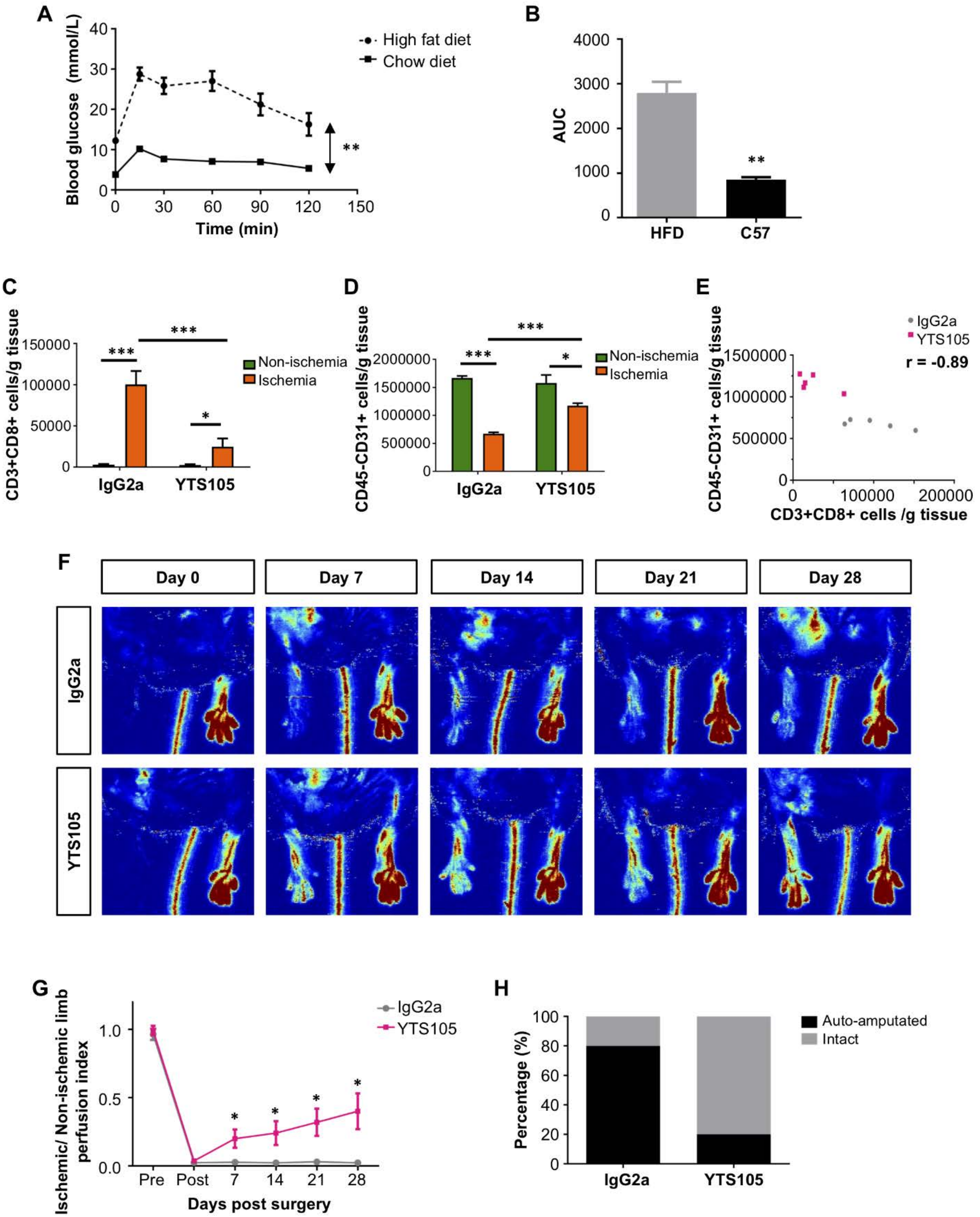

A
Blood glucose (mmol/L)
Time (min)
High fat diet
Chow diet
**
B
AUC
HFD
C57
**
C
CD3+CD8+ cells/g tissue
Non-ischemia
Ischemia
IgG2a
YTS105
D
CD45-CD31+ cells/g tissue
Non-ischemia
Ischemia
IgG2a
YTS105
E
CD45-CD31+ cells/g tissue
CD3+CD8+ cells /g tissue
IgG2a
YTS105
r = -0.89
F
Day 0
Day 7
Day 14
Day 21
Day 28
IgG2a
YTS105
G
Ischemic/ Non-ischemic limb perfusion index
Days post surgery
Pre
Post
IgG2a
YTS105
H
Percentage (%)
Auto-amputated
Intact
IgG2a
YTS105

Figure S2 CD8 checkpoint blockade increases vascular density in the ischemic tissues of DIO mice after injury. (A) Flow cytometric analysis and (B, C) quantification among $CD45^{-}CD31^{+}$ cells in the ischemic and non-ischemic muscles showing significantly increased $\%YFP^{+}CD31^{+}$ mature ECs and significantly reduced $\%YFP^{+}CD31^{-}$ immature ECs in the ischemic muscles of YTS105 than IgG2a-treated high fat diet-fed Cdh5-Cre;Rosa-YFP reporter mice. In this figure, all data are presented as mean +/- S.E.M, n=5 per group, *indicates $p<0.05$ and **$p<0.01$.

Figure S2

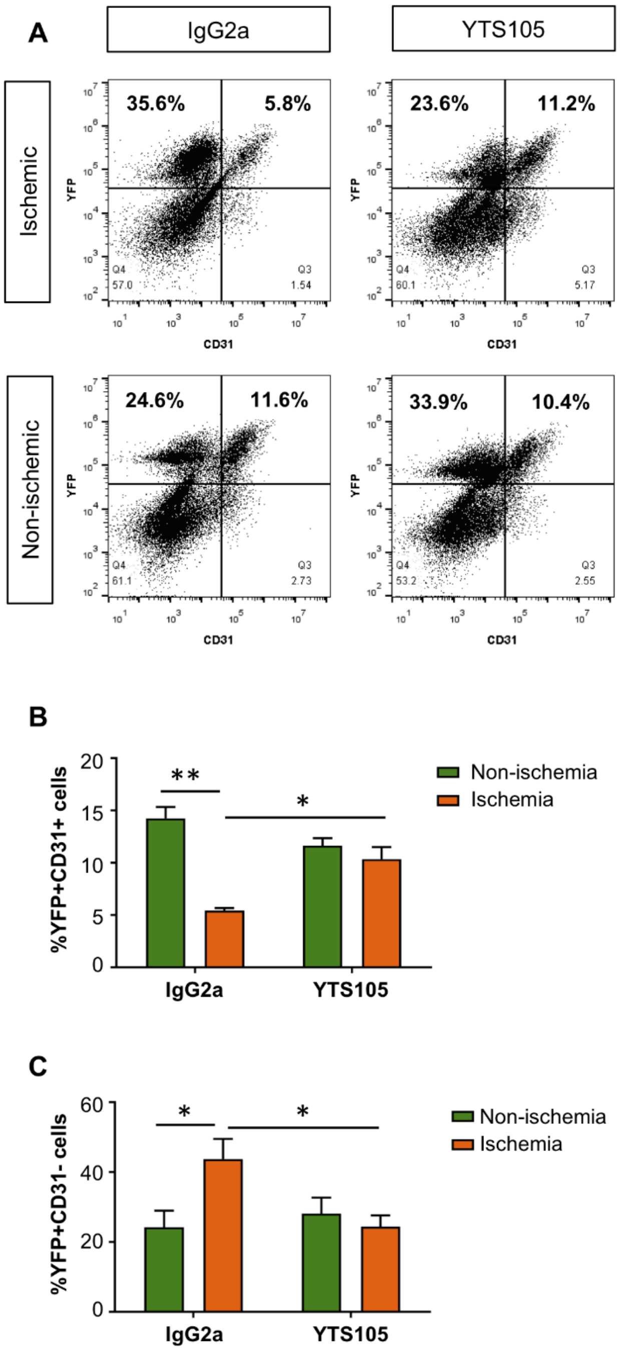
A
IgG2a
YTS105
Ischemic
Non-ischemic
35.6%
5.8%
23.6%
11.2%
24.6%
11.6%
33.9%
10.4%
YFP
CD31
Q4
57.0
Q3
1.54
Q4
60.1
Q3
5.17
Q4
61.1
Q3
2.73
Q4
53.2
Q3
2.55
B
%YFP+CD31+ cells
Non-ischemia
Ischemia
IgG2a
YTS105
C
%YFP+CD31- cells
Non-ischemia
Ischemia
IgG2a
YTS105

Figure S3 Genome-wide RNA-sequencing reveals the purity of $CD45^+CD3^+CD8^+$ T-cells sorted by flow cytometry. Approximately 1,000 $CD45^+CD3^+CD8^+$ cells were purified from the ischemic muscles of $Lepr^{db/+}$ and $Lepr^{db/db}$ mice, respectively, at day 7 after injury by flow cytometry. Biaxial scatter plots showing the relative expression levels of (A) *Cd3*- , (B) *Cd4*- or (C) *Cd8*-related transcripts by T-cells of all three distinct subsets on *t*-SNE plots.

Figure S3

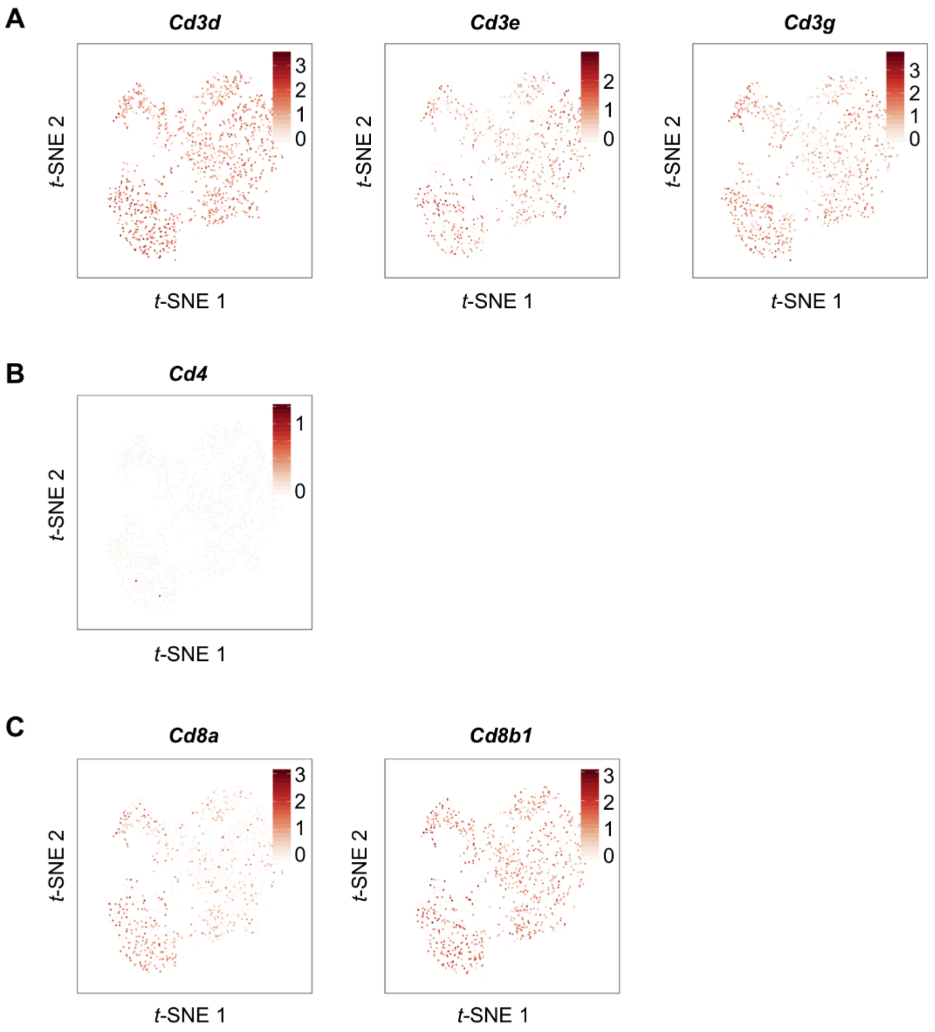

Figure S4 Genome-wide RNA-sequencing reveals the alternative cell fate commitment of CD8$^+$ T-cells of Lepr$^{db/+}$ and Lepr$^{db/db}$ mice after ischemic injury. Approximately 1,000 CD45$^+$CD3$^+$CD8$^+$ cells were purified from the ischemic muscles of Lepr$^{db/+}$ and Lepr$^{db/db}$ mice, respectively, at day 7 after injury by flow cytometry. (A) Monocle ordering of individual cells showing two branched developmental and response trajectories of CD8$^+$ T-cells of Lepr$^{db/+}$ and Lepr$^{db/db}$ mice after ischemic injury, respectively. (B) Branch-dependent genes are identified by BEAM analysis and four distinct clusters are further illustrated during fate 1 or 2 commitment of CD8$^+$ T-cells in Figure 5E. Here, GO enrichment analysis showing the top five most significant biological processes involved during cell fate commitment of CD8$^+$ T-cells as determined by the branch-dependent genes of the four clusters.

Figure S4

A

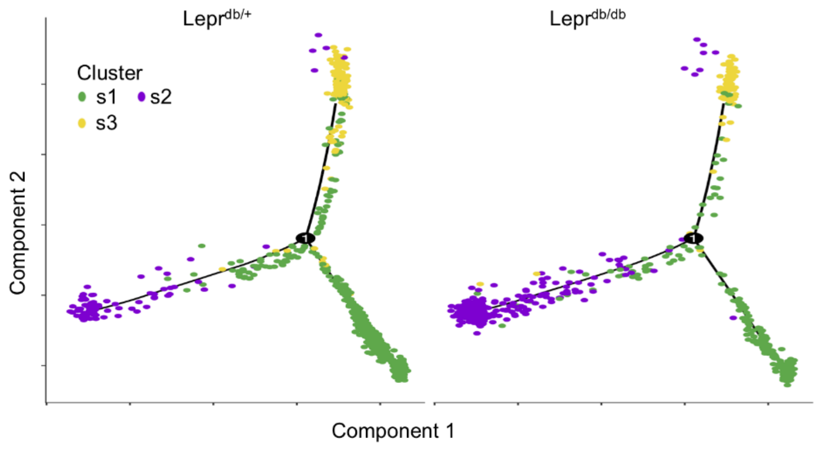

Lepr$^{db/+}$
Lepr$^{db/db}$
Cluster
s1
s2
s3
Component 2
Component 1

B

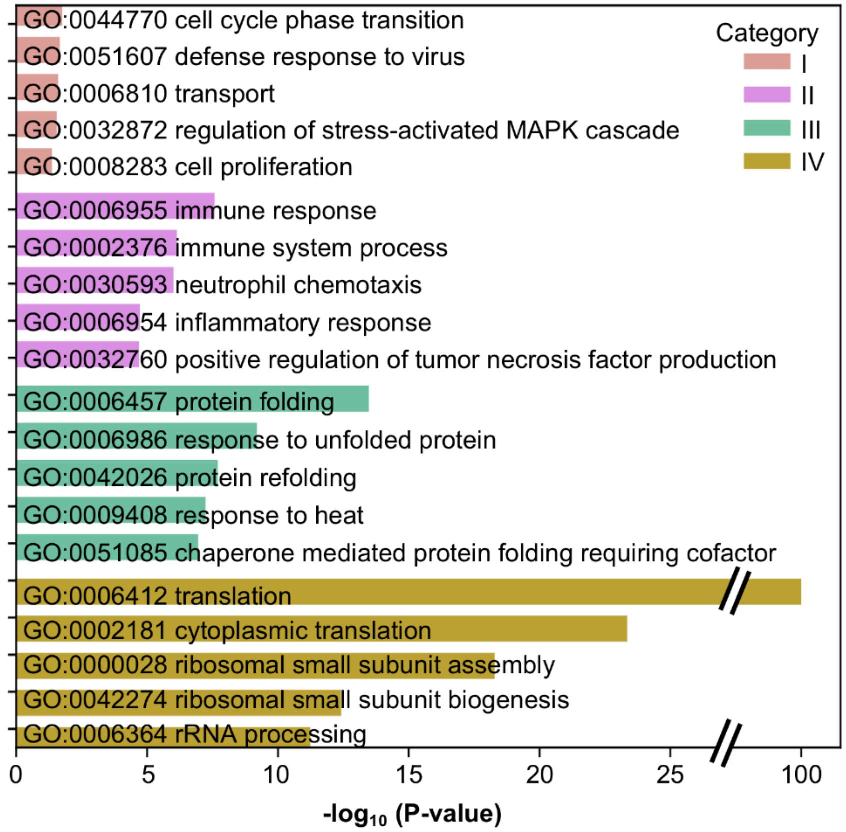

GO:0044770 cell cycle phase transition
GO:0051607 defense response to virus
GO:0006810 transport
GO:0032872 regulation of stress-activated MAPK cascade
GO:0008283 cell proliferation
GO:0006955 immune response
GO:0002376 immune system process
GO:0030593 neutrophil chemotaxis
GO:0006954 inflammatory response
GO:0032760 positive regulation of tumor necrosis factor production
GO:0006457 protein folding
GO:0006986 response to unfolded protein
GO:0042026 protein refolding
GO:0009408 response to heat
GO:0051085 chaperone mediated protein folding requiring cofactor
GO:0006412 translation
GO:0002181 cytoplasmic translation
GO:0000028 ribosomal small subunit assembly
GO:0042274 ribosomal small subunit biogenesis
GO:0006364 rRNA processing
Category
I
II
III
IV
0
5
10
15
20
25
100
-log$_{10}$ (P-value)

Figure S5 $CD8^+$ T-cells of the ischemic tissues of $Lepr^{db/db}$ mice express more cytotoxic granules and cytokines than that of $Lepr^{db/+}$ mice after injury. (A, B) Flow cytometric analysis showing the representative plots of $CD45^+CD3^+CD8^+$ cells with expression of specific cytotoxic granules and cytokines in the ischemic muscles of $Lepr^{db/+}$ and $Lepr^{db/db}$ mice, respectively, at day 14 after injury.

Figure S5

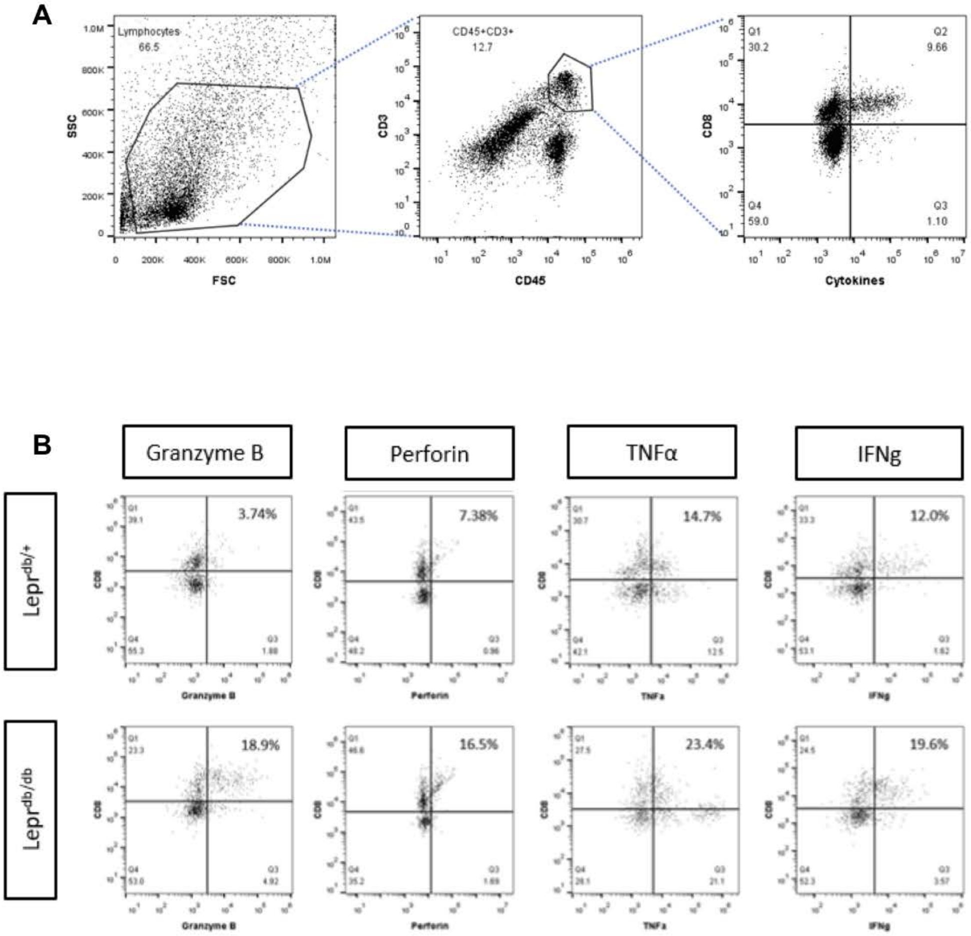
A
Lymphocytes
66.5
SSC
FSC
CD45+CD3+
12.7
CD3
CD45
Q1
30.2
Q2
9.66
Q4
59.0
Q3
1.10
CD8
Cytokines
B
Granzyme B
Perforin
TNFα
IFNg
Lepr^db/+
Lepr^db/db
3.74%
7.38%
14.7%
12.0%
18.9%
16.5%
23.4%
19.6%

Figure S6 CD8 checkpoint blockade reduces the expression of cytotoxic granules and cytokines by $CD8^+$ T-cells of the ischemic tissues of $Lepr^{db/db}$ mice after injury. (A, B) Flow cytometric analysis showing the representative plots of $CD45^+CD3^+CD8^+$ cells with expression of specific cytotoxic granules and cytokines in the ischemic muscles of IgG2a- and YTS105-treated $Lepr^{db/db}$ mice, respectively, at day 14 after injury.

Figure S6

A

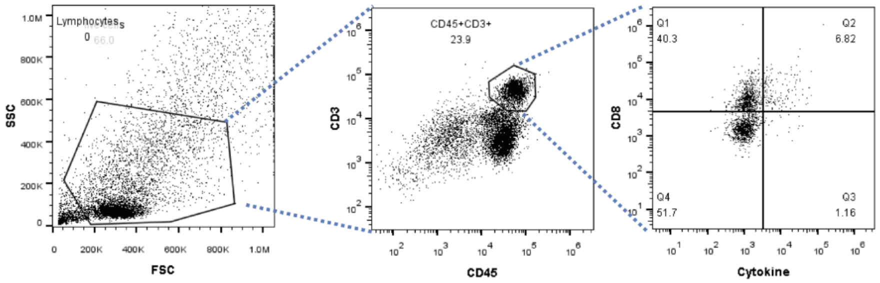
Lymphocytes
SSC
FSC
CD45+CD3+
23.9
CD3
CD45
Q1
40.3
Q2
6.82
Q4
51.7
Q3
1.16
CD8
Cytokine

B

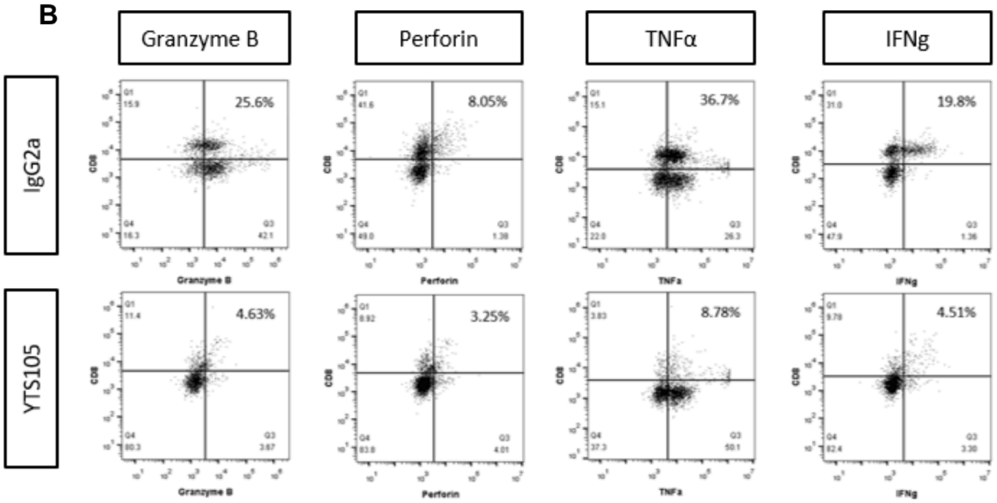
Granzyme B
Perforin
TNFα
IFNg
IgG2a
YTS105
25.6%
8.05%
36.7%
19.8%
4.63%
3.25%
8.78%
4.51%

Supplementary Tables

Table S1 A gene list of branch-dependent genes related to fate 1 or 2 commitment of CD8$^+$ T-cells in Lepr$^{db/+}$ and Lepr$^{db/db}$ mice. Branched-dependent genes are identified in the four distinct clusters of Figure 5E by BEAM analysis.

**Clusters**	**Genes**
**I**	Map2k1, Rbbp7, Prdx1, Rbx1, Tmod3, Man1b1, Tapbp, Mfap1a, Tmem9b, Bcas2, Serpinb1a, Polr3k, Snx17, Ppp2r4, Ldha, Ndufa11, H2afz, Srsf5, Ndufa4, Uqcrb, Fam107b, Atp5a1, Ube2d2a, Oaz1, Ppia, Eif1, Sar1a, Tnfrsf18, Ramp3, Ftl1, Epb41, Zc3hav1, Gpx4, Fosb, Lsm1, Ywhaq, Uba2, Eif4a2, Ppp4c, Vps37b, Psmc1, Klf13, Whsc1l1, Prelid1, Cytip, Bnip3l, Tnrc6b, Slc25a3, Samsn1, Gramd3, Psmb2, Pdcd6, Rrp1, Stat3, Maf1, Jak1, Hsbp1, Gpr132, Pitpnc1, Ier3ip1, Kmt2e, Isca1, Brk1, Ankrd11, Atp5d, Pomp, Ptprc, Ube2l3
**II**	Ikzf2, Cd74, Mylpf, Cd14, Lyz2, Fcer1g, Trf, Apoe, Cd63, Acta1, Tnnc1, Fcgr3, Polk, H2-Ab1, Pdcd4, Spp1, Spry2, Adora2a, Lamtor4, Alg5, Ubl5, Hnrnpf, Spcs1, Ost4, Morf4l1, Pld3, Mpc1, Crem, Ninj1, Emb, Tax1bp1, Cirbp, Aamp, Selplg, Snx4, Fyb, Cox5a, Prdx6, Cdk2ap2, Uqcrc1, Gps2, Gimap5, Rabac1, Shfm1, Arpc3, Chmp4b, Napa, Cenpa, Dock10, Pde2a, 1700025G04Rik, Rnf138, Park7, Hsp90b1, Tbcb, Cox6b1, B4galnt1, Atp6v1f, Sqstm1, Plac8, Gimap4, Nfkb2, Gabarapl2, Gnb1, Arpc1b, Ifngr1, Mif4gd, Hn1, Lamtor5, Ptpn22, Cd28, Vamp4, Setd3, Naa35, Ergic2, Ubc, Gmfg, Ift20, Dennd4c, Gimap6, Rwdd1, Tor2a, Ap3s1, Necap2, Sdhb, Gimap3, Bcap31, Rab8a, Prkca, Ap2s1, Anxa7, Cnih4, Dazap1, Pitpna, Arl6ip5, Ly6a, Cnn2, Plp2, Ywhah, Aplp2, 5031425E22Rik, Tmed2, Tpm4, Fam134c, Tbcc, Rtf1, Arhgdia, Ngdn, Eif3g, Ap2m1, Sin3b, Letm1, Tpm3-rs7, Pon2, Ms4a6b, Snapc5, S100a13, Cers2, Pdcd1, Ifitm1, Klrg1, Gskip, Zeb2, Esm1, Impa2, Fxyd5, Smim3, Arf1, Actg1, Ubald2, Gbp7, Pnisr, Prpsap1, Ppp1cc, Mat2b, Rnf14, Glipr2, Csnk2b, Plcl2, Irf2, Vamp8, Prkcz, Tmem50a, Camk4, Slfn2, Vasp, Sh2d1a, Ccl5, Cyba, Cd44, Rgs1, Il18r1, Cd48, Sub1, Ltb, S100a4, Nkg7, Vim, H2-Q7, Myl12a, Ric1, S100a10, Supt4a, Tgfb1, Ahnak, Icos, Malat1, Sh3bgrl3, Stx11, Cx3cr1, Gzmk, Ccr2, Osbpl3, Eno1, Cyth4, Dok2, Ndfip2, Cnbp, Shisa5, Alcam, Hmgb2, Hist1h1c, Id2, Bhlhe40, Rac2, Higd1a, Sp100, Hcst, Cotl1, Calm2, Cxcr6, Bcl2a1d, Lgals3, Tnfrsf9, Bcl2a1b, Klrc1, Itgb1, Serpinb9, Gzma, Cxcr3, Sytl3, Fhl2, Fasl, Dusp2, B2m, Slc3a2, Klre1, Lgals1, Rora, Gzmb, S100a6, Ifng, Ccl4, Ccl3, Pfn1, Cd52, Ctla2a, Tmsb4x, Terf2ip, Pde4d, Tmem59, Edf1, Ndufa8, Gramd1a, Ube2i, Dusp1, Map2k2, Dek, Atp5j2, D16Ertd472e, Slc25a5, Arpp19, Arpc5, Eif6, Esd, Cox17, Laptm5, Armc7, Hnrnpa2b1, Pxn, Tnfaip3, Stat4, Capzb, Zap70, Ppp1r12a, Raly, Rdx, Ctsb, Dusp5, Sult2b1, Abcb1a, Spcs2, Plekha2, Uqcr11, Cks2, Ptp4a2, Atp5j, Ybx3, Dbf4, Smox, Gimap1, Rbm39, Pafah1b1, Sdcbp2, Sp110, Tnfrsf1b, H2-T22, Sumo2, Ccnc, Rap1b, Rnf216, Itgal, Sh2d2a, Anxa6, Aldoa, Mapkapk3, Psmb8, Arhgap26, Lpxn, Gimap7, Cd6, Glrx, Plek, Dynlrb1, Baiap2, Map2k3, Ctla4, Bet1l, Syf2, Psmc4, Calm1, Fkbp1a, Ddx5,

	1110008P14Rik, Cpt1a, Akap13, Pdia3, Psmb3, Stmn1, Abcb1b, Gbp3, Kcnj8, Acadl, Cd47, Gna15, Lilrb4a, Lig1, Gm19585, Ppig, Grap2, Fryl, Eif4h, Rbms1, Ypel3, Hadhb, Tmem123, Dad1, Fmnl1, Ostf1, Ppp1r16b, Itm2b, Tpst2, Reep5, Dnajb6, Txn1, Pttg1, Cdc37, Selk, Fam103a1, Fth1, Cmip, Gnai2, Arf5, AW112010, Serpinb6b, Prr13, Plekhb2, Il10rb, Ccl9, Rpa2, Zyx, Anxa2, Socs2, Nr3c1, Klrc2, Cdc42ep3, Ndfip1, Litaf, Mt1, Slc9a3r1, Pik3r1, Atp2b4, Il18rap, Lilr4b, Klrk1, Arl6ip1, Klrd1, Rnf166, Serinc3, S100a11, Rab8b, Mkrn1, H3f3b, Hnrnpa3, Clic1, Frg1, BC004004, Celf2, Myl6, Itm2c, Jakmip1, Cd38, Iscu, Vps29, Prkch, Ets1, Cd53, Dnajc8, Arpc2, H2-K1, Cd2, Gdi2, Crip1, Prkar1a, Il12rb2, Arpc4, Gnb2, Cd3d, Clta, Srgn, Ankrd44, Hnrnpl, Ube2d3, Dgat1, Rnaset2a, Ms4a4b, Szrd1, H2-D1, Cd82, Tmbim6
**III**	Chchd2, Sec62, Slc38a2, Gadd45g, Ccnd2, Zfand2a, Arrdc4, Mcl1, Id3, Pcbp1, Mapk6, Swt1, Vps8, Amd1, Gprasp1, Hspa4l, St13, Tsc22d1, Map3k8, Ppp1r15a, Plekho1, 1500011K16Rik, Metap1d, Zfp36l1, Jun, Myc, Auts2, Fas, Cdkn2d, Hspe1, Ddit4, Dnajb4, Hsp90aa1, Hsph1, Alkbh1, Hsp90ab1, Hspd1, Fkbp4, Tmsb10, Cd69, Cacybp, Gadd45a, Rhoh, Ahsa1, Gprasp2, Nr4a1, Hspa1a, Hspa8, Pmaip1, Dnajb1, Arl4a, Hspa1b, Bag3, Ypel2, Gm7120, Ccdc117, Atf3, Prickle1, Herpud1, Hspa2, Cdr2, Hspb1, Slc25a25, Hotairm1, Tnfsf8, Lman2l, Igtp, Xcl1, Dnaja4, Phlda1, Dnaja1, Tra2b, Klf6, Gm8797, Ubb
**IV**	Wdr91, Rgcc, Treml2, Dph5, Rpl10-ps3, Nsg2, Ccr9, Nme1, Rragd, Tmem108, Enpp4, Ppcdc, Gm10827, P4ha1, Igfbp4, Apex1, Fdx1l, Dapl1, Nop58, Pigp, Bcl2, Tra2a, Skp1a, Mast4, mt-Co1, mt-Atp6, mt-Co3, mt-Cytb, Tspan13, mt-Nd3, mt-Nd4, mt-Nd1, Fos, Bola2, mt-Nd2, Rps11, Itm2a, Loxl2, Ifngr2, Npc2, Atp11b, Smchd1, Tdrp, Dusp10, Gm43698, Txk, Ccr7, Klhdc1, Rpl41, Lef1, Rps6, Gm10073, Gm10076, Rpl4, Rpl8, Rpl15, Tpt1, Eef1a1, Jmjd1c, Gm6133, Rpl36-ps3, Gm10036, Tubb2b, Rpl6l, Pik3ip1, Uba52, Rpl22l1, Rpl10, Rpl13-ps3, Rpl9-ps6, Rps27rt, Rps27, Rps29, Rps3, Rpl35a, Rps7, Rps14, Gm8730, Rplp0, Rpl30, Rpl9, Rps3a1, Gm9493, Wdr89, Rpl11, Gm11808, Eef1g, Rps12-ps3, Rpl23a-ps3, Rps10, Rpl29, Rps26, Rpl36, Rpl38, Rpl39, Rpl27a, Rpl32, Rps15, Rpl22, Rps26-ps1, mt-Atp8, Rps13, Rpsa, Rpl26, Rpl34, Socs3, Rpl31, Rps2, Rpl13, Rps5, Rpl3, Rpl18a, Rps27a, Rps9, Rps17, Rps15a, Rpl5, Rpl36a, Rps20, Rpl37a, Rps19, Rpl35, Rps16, Rps23, Rpl23a, Rpl10a, Rps12, Rpl28, Gm10260, Rps18-ps3, Rpl23, Rps18, Eef1b2, Rps24, Rpl17, Rpl21, Rplp2, Rps21, Rpl37, Rps28, Rpl12, Rps25, Rpl6, Rplp1, Rps4x, Rps8, Rpl14, Actn1, Gnb2l1, Fam101b, Gm2000, Rpl18, Rpl19, Tubb2a, Rgs10, Sell

Table S2 GO enrichment analysis showing the branch-dependent genes of the top five most significant biological processes involved during cell fate commitment of CD8^{+} T-cells. Branched-dependent genes are identified in the four distinct clusters of Figure 5E by BEAM analysis.

**Clusters**	**P value**	**GO Term**	**Genes**
**I**	0.017574254	GO:0044770~cell cycle phase transition	PTPRC, UBE2L3
	0.021373691	GO:0051607~defense response to virus	PTPRC, POLR3K, ZC3HAV1, BNIP3L
	0.024690841	GO:0006810~transport	ATP5D, NDUFA4, RAMP3, PRELID1, OAZ1, SNX17, SLC25A3, VPS37B, PITPNC1, ATP5A1, SAR1A, NDUFA11, UQCRB
	0.0279725	GO:0032872~regulation of stress-activated MAPK cascade	MAP2K1, PRDX1
	0.043062319	GO:0008283~cell proliferation	MAP2K1, UBE2L3, PRDX1, STAT3
**II**	2.66E-08	GO:0006955~immune response	FYB, H2-K1, CCL3, CCL9, CTLA4, H2-D1, MYLPF, H2-AB1, CCL5, CCL4, FTH1, CD74, B2M, BCAP31, SERPINB9, TNFRSF9, TNFRSF1B, CCR2, IFNG, ZAP70, FASL, LTB, CD28
	7.31E-07	GO:0002376~immune system process	H2-K1, HMGB2, IFITM1, LGALS3, LILRB4A, KLRK1, H2-D1, CTLA4, PTPN22, H2-AB1, SP110, H2-Q7, PSMB8, CD74, PDCD1, B2M, SERINC3, ALCAM, SH2D1A, KLRG1, CAMK4, ETS1, SQSTM1, ZAP70, CD14
	9.83E-07	GO:0030593~neutrophil chemotaxis	PRKCA, CCL3, LGALS3, IFNG, CCL9, FCER1G, PDE4D, CCL5, CCL4, FCGR3, SPP1
	1.89E-05	GO:0006954~inflammatory response	PRKCZ, CCL3, HMGB2, MAP2K3, CCL9, NFKB2, CXCR3, CCL5, CCL4, TGFB1, PARK7, CYBA, TNFRSF9, TNFRSF1B, CAMK4, CCR2, CXCR6, ZAP70, TNFAIP3, CD14, SPP1

	2.01E-05	GO:0032760~positive regulation of tumor necrosis factor production	CYBA, CCL3, SELK, IFNG, CD2, FCER1G, CCL4, PIK3R1, CD14
**III**	3.33E-14	GO:0006457~protein folding	HSP90AB1, HSP90AA1, FKBP4, ST13, DNAJA1, HSPA4L, HSPE1, HSPD1, DNAJB1, DNAJB4, DNAJA4, AHSA1, HSPA8
	6.35E-10	GO:0006986~response to unfolded protein	HSPH1, HERPUD1, HSP90AA1, HSPA4L, HSPB1, HSPE1, HSPA1A, HSPD1
	2.01E-08	GO:0042026~protein refolding	HSP90AA1, HSPA2, HSPD1, DNAJA4, HSPA8
	5.88E-08	GO:0009408~response to heat	HSP90AA1, HSPA2, DNAJA1, HSPA1A, HSPD1, HSPA1B, DNAJA4
	1.13E-07	GO:0051085~chaperone mediated protein folding requiring cofactor	HSPH1, HSPE1, DNAJB1, HSPD1, HSPA8
**IV**	6.17E-103	GO:0006412~translation	RPL18, RPL17, RPL36A, RPL19, RPL14, RPL13, RPL15, RPS18-PS3, RPL22L1, RPS2, RPS3, RPL10, RPL11, RPL12, RPS27A, RPL35A, RPS4X, RPS18, RPS19, RPL41, RPS16, RPS17, GM6133, RPS14, RPS15, RPS12, RPS13, EEF1G, RPS11, UBA52, EEF1B2, RPL27A, RPL35, RPL9-PS6, RPL36, RPS15A, RPL37, RPL38, RPL39, RPS26, RPS27, GM10036, RPL30, RPS28, RPS29, RPL32, RPL6, RPL31, RPL34, RPL9, RPL8, RPL3, RPL5, RPL10A, RPS20, RPL4, RPS27RT, RPS21, RPS23, RPS24, RPL23A-PS3, RPSA, EEF1A1, RPL13-PS3, RPL26, GM10260, RPS9, RPL23A, RPS6, RPL36-PS3, RPS5, RPS8, RPL28, RPS7, RPS3A1, RPL29, RPL23, GM9493, RPL18A, RPL22, RPL21, RPL37A
	4.48E-24	GO:0002181~cytoplasmic translation	GM10073, RPL35A, RPL6L, RPL15, RPL26, RPL36, RPLP2, RPL22L1, RPL29, RPL31, RPL6,

			RPL22, RPL9, RPLP0, RPLP1, RPL8
	5.21E-19	GO:0000028~ribosomal small subunit assembly	RPS25, RPSA, RPS27, RPS19, RPS28, RPS17, RPS14, RPS15, RPS10, RPS27RT, RPS2, RPS5
	3.81E-13	GO:0042274~ribosomal small subunit biogenesis	RPS19, RPS28, GM9493, RPS16, RPS17, RPS15, RPS6, RPS24, RPS7
	5.79E-12	GO:0006364~rRNA processing	RPL35A, RPL14, RPL26, RPS6, RPS7, RPS28, RPS19, GM9493, RPS16, RPS17, RPS15, RPL5, RPL11, RPS24
